# Global burden of zoonotic infectious diseases of poverty, 1990–2021

**DOI:** 10.1186/s40249-024-01252-x

**Published:** 2024-11-06

**Authors:** Chao Lv, Yiwen Chen, Zile Cheng, Yongzhang Zhu, Weiye Chen, Nan Zhou, Yiming Chen, Yinlong Li, Wangping Deng, Xiaokui Guo, Min Li, Jing Xu

**Affiliations:** 1 School of Global Health, Chinese Center for Tropical Diseases Research, Shanghai Jiao Tong University School of Medicine, Shanghai, 200025 China; 2 National Institute of Parasitic Diseases, Chinese Center for Disease Control and Prevention (Chinese Center for Tropical Diseases Research), National Health Commission Key Laboratory of Parasite and Vector Biology, National Key Laboratory of Intelligent Tracking and Forecasting for Infectious Diseases, WHO Collaborating Centre for Tropical Diseases, National Center for International Research On Tropical Diseases, Shanghai, China; 3 School of Public Health, Shanghai Jiao Tong University School of Medicine, Shanghai, China

**Keywords:** Global burden of disease, Schistosomiasis, Cystic echinococcosis, Cysticercosis, Food borne trematodiases, Disability-adjusted life year, Prevalence, Age-standardized rate

## Abstract

**Background:**

The zoonotic infectious diseases of poverty (zIDPs) are a group of diseases contributing to global poverty, with significant impacts on a substantial population. This study aims to describe the global, regional, and national burden of zIDPs—schistosomiasis, cystic echinococcosis, cysticercosis, and food-borne trematodiases (FBTs)—to support policy making and resource allocation for their control and elimination.

**Methods:**

Data of zIDPs from the Global Burden of Diseases, Injuries, and Risk Factors Study (GBD) 2021 were retrieved from 1990 to 2021. The age-standardized prevalence rate (ASPR), age-standardized mortality rate (ASMR), and age-standardized disability-adjusted life-year (DALY) rate were described and the estimated annual percentage changes (EAPCs) were calculated to quantify their burden and temporal trends. Spearman correlation analysis was conducted to examine the relationship between age-standardized rates and Socio-demographic Index (SDI).

**Results:**

In 2021, these zIDPs exhibited a certain level of ASPRs and age-standardized DALY rates, while maintaining relatively low ASMRs. Noticeably, schistosomiasis presented the highest ASPR of 1914.299 (95% UI: 1378.920, 2510.853 per 100,000 population) and an age-standardized DALY rate of 21.895 (95% UI: 12.937, 37.278 per 100,000 population) among the zIDPs. The tapestry of burden—woven predominantly through low and lower-middle SDI regions—stretched across Africa, Latin America, and parts of Asia. From 1990 to 2021, a kaleidoscopic shift was observed globally as ASPRs, ASMRs, and age-standardized DALY rates declined significantly, as reflected by the EAPC values. Negative correlations were observed between the ASPRs, ASMRs, age-standardized DALY rates of schistosomiasis (r value = − 0.610, − 0.622 and − 0.610), cystic echinococcosis (− 0.676 of ASMR, − 0.550 of age-standardized DALYs), cysticercosis (− 0.420, − 0.797 and − 0.591) and the SDI. In contrast, a slight positive correlation was noted between the ASPR, age-standardized DALY rates of FBTs and SDI with r value of 0.221 and 0.213, respectively.

**Conclusion:**

The burden of zIDPs declined across almost all endemic regions from 1990 to 2021, yet still predominated in low and low-middle SDI regions. Substantial challenges exist to achieve the goal of control and elimination of zIDPs, and integrated approaches based on One Health need to be strengthened to improve health outcomes.

**Graphical Abstract:**

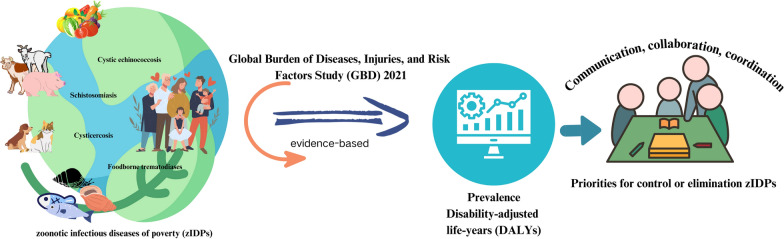

**Supplementary Information:**

The online version contains supplementary material available at 10.1186/s40249-024-01252-x.

## Background

Infectious diseases of poverty (IDPs) are a group of infectious diseases predominantly thriving in conditions of poverty and socioeconomic inequality, with considerably impacts on public health [1, 2]. These diseases encompass, but are not limited to malaria, AIDS, tuberculosis, dengue fever, and neglected tropical diseases (NTDs). IDPs are dominant in low- and middle-income countries, particularly in sub-Saharan Africa, South Asia, Southeast Asia, and Latin America [3], characterized by lack of basic sanitation facilities, clean drinking water, and primary healthcare. Additionally, overcrowded living conditions with poor hygiene and sanitation facilitated the spread of these diseases [4, 5]. Poverty further restricts individuals’ ability to access essential resources for the prevention and treatment of these diseases, including vaccines, diagnostic tools and medications [6]. IDPs also hamper the social and economic development, resulting in loss of labor force, decreased productivity, and increased financial stress on families and communities [1, 4, 6]. The significant disease burden in these regions makes them more challenging to escape from poverty, perpetuating a vicious cycle.

Among the IDPs, schistosomiasis, cystic echinococcosis, cysticercosis, food-borne trematodiases (FBTs) comprising opisthochiais, clonorchiasis, paragonimiasis, fascioliasis, fasciolopsis, etc., are a group of zoonotic diseases with multiple hosts and complicated transmission routes, posing greater challenge to be controlled or eliminated [7]. Schistosomiasis, also known as bilharzia, is an acute and chronic disease caused by blood flukes (trematode worms) of the genus *Schistosoma*. It is ranked second place in parasitic diseases harmful to humans after malaria, being prevalent in 78 countries and territories worldwide with more than 200 million people requiring preventive chemotherapy and about 800 million people at risk of infection [8]. Cystic echinococcosis, also known as hydatid disease, is caused by the infection of the larvae of *Echinococcus granulosus*. It is globally distributed in every continent except Antarctica [9]. Cysticercosis is a parasitic infection caused by the larvae of the tapeworm *Taenia solium*. Infections occur though the consumption of tapeworm eggs, which hatch and develop into larvae, and then penetrate the host’s intestinal wall and migrate to tissues such as muscle, eyes, or brain. Populations at-risk are globally distributed, with higher infection rates in developing regions of Africa, Asia, and Latin America [10]. FBTs include a group of parasitic diseases of opisthochiais, clonorchiasis, paragonimiasis, fascioliasis and fasciolopsis. Humans become infected through the consumption of contaminated food, such as raw fish, crustaceans or vegetables, leading to severe clinical symptoms. FBTs are most prevalent in East Asia and South America with zoonotic and emerging nature [11]. Being a group of wild distributed parasitic diseases impacting on human beings, the World Health Organization (WHO) involved these zoonotic IDPs (zIDPs) in the “Ending the neglect to attain the Sustainable Development Goals: a road map for neglected tropical diseases 2021–2030”, aiming to eliminate schistosomiasis as a public health problem, and control cystic echinococcosis, cysticercosis and FBTs [12]. Understanding the burden and tendency of these zIDPs on humans are necessary for designing intervention programs and allocating limited resources to combat against zIDPs [13–15].

The Global Burden of Diseases, Injuries, and Risk Factors Study (GBD), led by the Institute for Health Metrics and Evaluation (IHME) at the University of Washington stands as the most comprehensive research initiative on disease burden and remains an invaluable global health data resource [16, 17]. Since its inception in the early 1990s, GBD has consistently provided systematic and comprehensive estimates of global health and health loss, stratified by age groups, locations, and sexes [18]. The availability of these estimates enables evidence-based global health assessments, facilitating the identification of health disparities and guiding the formulation and implementation of targeted interventions. Moreover, the GBD database serves as critical foundation for global health research, promoting extensive research underpinning disease burden analysis, health risk assessment, and evaluations of intervention effectiveness [19]. GBD 2021 is an update of the 2019 data, emphasizing emerging and existing health threats that are prioritized on the international public health agenda. This is particularly significant in the context of COVID-19, which has transitioned from an urgent new threat to an infectious disease requiring long-term management and population coexistence [20]. In this study, the epidemiological features and burden of zIDPs are described using data from GBD 2021. The impacts of the factors such as the Socio-demographic Index (SDI), geographic location, gender, and age on diseases burden are also analyzed. The findings from this research not only enhance our understanding of the impacts of these zIDPs on humans, but also provide reference for guiding precise interventions and reasonable resource allocation.

## Methods

### The definition of zIDPs

The WHO's list of NTDs includes 21 diseases closely associated with poverty, such as schistosomiasis, FBTs, cysticercosis, and echinococcosis. According to Zhou’s classification [7], schistosomiasis, opisthorchiasis, clonorchiasis, paragonimiasis, fascioliasis, fasciolopsiasis, cysticercosis, and echinococcosis are categorized as zoonotic, ancient diseases. Furthermore, in both the NTDs list and the GBD database, FBTs encompass diseases such as opisthorchiasis, clonorchiasis, paragonimiasis, fascioliasis, and fasciolopsiasis. Based on this classification, we define zoonotic infectious diseases of poverty (zIDPs) as a group diseases including schistosomiasis, cystic echinococcosis, cysticercosis, and FBTs in this study.

### Data source and extraction

GBD 2021 provided estimates of the worldwide burden of 371 diseases and injuries across 204 nations and territories from 1990 to 2021. These data were accessible via the Global Health Data Exchange (GHDx) results tool (http://ghdx.healthdata.org/gbd-resultstool), and were obtained through their official website using a web browser [16, 17].

An important index used in GBD 2021 was the SDI, a comprehensive indicator reflecting the social and economic conditions that influence health outcomes in each location. SDI is calculated based on the total fertility rate for those under 25 years old, the mean education level for individuals aged 15 and older, and lag-distributed income per capita. For GBD 2021, SDI values were scaled from 0.00 to 1.00, categorizing countries and territories into five development levels as followings: low (< 0.46), low-middle (0.46–0.60), middle (0.61–0.69), high-middle (0.70–0.81), and high (> 0.81).

The specific search criteria in the “Search” interface were as follows: GBD Estimate (Cases of death or injury, risk factor), Measure [Prevalence, Deaths, Age-standardized disability-adjusted life-years (DALYs)], Metric (Number, Percent, Rate), Cause (schistosomiasis, cystic echinococcosis, cysticercosis, FBTs), Location (Global, All countries and regions, Different SDI regions, 204 countries and territories), Age (All ages, Age-standardized, < 5 years, 5–9 years, 10–14 years, 15–19 years, 20–24 years, 25–29 years, 30–34 years, 35–39 years, 40–44 years, 45–49 years, 50–54 years, 55–59 years, 60–64 years, 65–69 years, 70–74 years, 75–79 years, 80–84 years, 85–89 years, 90–94 years, 95+ years), sex (both, male, female), year (1990–2021, and each year from 1990 to 2021).

The extraction dataset of zIDPs included quantified indicators of case numbers, age-standardized prevalence rates (ASPRs), death numbers, age-standardized mortality rates (ASMRs), DALYs, and age-standardized DALYs rates at level of globe, SDI regions, GBD regions and across all age groups from 1990 to 2021. The ASPR, ASMR and age-standardized DALYs rate were calculated using the following formula:

$$ASR = \frac{{\sum\nolimits_{i = 1}^{N} {a_{i} w_{i} } }}{{\sum\nolimits_{i = 1}^{N} {wi} }}$$

where α_*i*_ is the age-specific rate in the $i$th age group and $w_{{\text{i}}}$ represents the number of person(or the weight) in the same age group among the GBD standard population. *N* is the number of age groups. 95% uncertainty intervals (UIs) were defined as the 2.5th and 97.5th values of the ordered 1000 draws. There was no significant difference between numerical values if their UIs overlapped.

### Statistical analysis

The estimated annual percentage changes (EAPCs) in ASRs from 1990 to 2021 were calculated to describe the fluctuation trends of the burden of zIDPs. EAPCs and their 95% confidence intervals (*CI*s) were used to identify trends over specific time intervals. A statistically significant declining trend was indicated when the upper limit of the EAPC (95% *CI*) was below zero. Conversely, a statistically significant increasing trend was defined when the lower limit of the EAPC (95% *CI*) exceeded zero. If the EAPC (95% *CI*) included zero, the change in ASRs was considered without statistical significance.

Smoothing spline models were employed to assess the relationship between the indicators of ASPR, ASMR, and age-standardized DALYs rate of zIDPs and the SDI across 21 GBD regions, as well as 204 countries and territories. The smooth splines were fitted using the Locally Weighted Scatterplot Smoothing (LOWESS) method, which automatically determines the degree, number, and location of knots based on the data and the span parameter [16, 17]. Spearman correlation analysis was conducted to estimate the R indices and *P* values for the association between ASR and SDI, with *P* < 0.05 considered statistically significant.

All analyses were performed using R software (version 4.2.3; R Foundation for Statistical Computing, Vienna, Austria, https://cran.r-project.org/).

## Results

### The prevalence and temporal trend of zIDPs

#### The prevalence burden of schistosomiasis

Globally, an estimated 151.377 million (95% UI: 109.062, 198.666 million) people were affected by schistosomiasis in 2021, with an ASPR of 1914.299 per 100,000 population (95% UI: 1378.920, 2510.853) (Table S1, Table 1). The ASPR of schistosomiasis showed a decline trend according to the EAPC (− 0.226, 95% *CI:* − 0.327, − 0.129) globally from 1990 to 2021. By SDI analysis, the low SDI region had the highest number of schistosomiasis cases (63.427 million, 95% UI: 45.057, 84.145 million) and the highest ASPR (5832.767 per 100,000 population, 95% UI: 4227.859, 7675.456), while the high SDI region reported the fewest cases and the lowest ASPR (Table S1, Table 1). The ASPR showed an annual downward trend from 1990 to 2021 across all SDI regions except the high SDI region (Fig. S1). The EAPC was − 0.537 (95% *CI*: − 0.592, − 0.485) in the low SDI region, showing the most significant decrease trend. By region analysis, schistosomiasis cases were reported in 10 out of the 21 GBD regions in 2021. Western sub-Saharan Africa had the highest number of cases (61.535 million, 95% UI: 44.035, 81.338 million), followed by Eastern sub-Saharan Africa (46.949 million, 95% UI: 33.903, 61.049 million) and East Asia (11.460 million, 95% UI: 8.954, 15.054 million). Western sub-Saharan Africa also recorded the highest ASPR, with 13,433.552 cases per 100,000 population (95% UI: 9651.799, 17,812.075), followed by Eastern sub-Saharan Africa (11,617.619, 95% UI: 8557.990, 15,093.062) and Central sub-Saharan Africa (7947.466, 95% UI: 5702.598, 11,151.884) (Table 1). Furthermore, the ASPR of schistosomiasis decreased across all 10 GBD regions from 1990 to 2021, except in the Caribbean. The highest EAPC was observed in North Africa and the Middle East, with a reduction of − 0.833 (95% *CI*:− 0.876, − 0.776) (Table 1).

**Table 1:** Age-standardized prevalence rate of zoonotic infectious diseases of poverty in 1990 and 2021, and estimated annual changes from 1990 to 2021

Regional classification	Schistosomiasis		Cystic echinococcosis		Cysticercosis		Food-borne trematodiases	
	ASPRs per 100,000, 2021 (95% UI)	EAPCs, 1990 to 2021 (95% *CI*)	ASPRs per 100,000, 2021 (95% UI)	EAPCs, 1990 to 2021 (95% *CI*)	ASPRs per 100,000, 2021 (95% UI)	EAPCs, 1990 to 2021 (95%* CI*)	ASPRs per 100,000, 2021 (95% UI)	EAPCs, 1990 to 2021 (95% *CI*)
Global	1914.299 (1378.920, 2510.853)	− 0.226 (− 0.327, − 0.129)	7.691 (6.269, 9.507)	0.044 (− 0.025, 0.100)	51.269 (37.166, 67.279)	− 0.251 (− 0.290, − 0.197)	526.740 (473.703, 593.254)	− 0.501 (− 0.527, − 0.469)
SDI regions								
High SDI	59.474 (28.775, 94.507)	0.963 (0.449, 1.626)	1.675 (1.299, 2.174)	0.252 (− 0.205, 0.298)	36.979 (26.456, 49.619)	0.035 (− 0.038, 0.120)	227.959 (207.689, 253.154)	− 0.104 (− 0.166, − 0.027)
High-middle SDI	123.113 (84.591, 172.950)	− 0.427 (− 0.533, − 0.329)	9.880 (8.115, 12.101)	− 0.104 (− 0.158, − 0.039)	38.506 (27.217, 52.351)	− 0.376 (− 0.418, − 0.319)	1232.730 (1094.866, 1404.875)	− 0.242 (− 0.288, − 0.190)
Middle SDI	1413.613 (1029.409, 1822.780)	− 0.169 (− 0.258, − 0.099)	7.578 (6.092, 9.404)	0.395 (− 0.341, 0.439)	59.619 (43.885, 77.928)	− 0.253 (− 0.293, − 0.205)	749.039 (675.542, 842.620)	− 0.633 (− 0.653, − 0.611)
Low-middle SDI	2461.033 (1805.468, 3212.180)	− 0.357 (− 0.455, − 0.261)	8.361 (6.852, 10.294)	− 0.059 (− 0.168, − 0.003)	62.060 (46.586, 78.066)	− 0.322 (− 0.370, − 0.260)	79.315 (65.261, 95.419)	− 0.453 (− 0.521, − 0.381)
Low SDI	5832.767 (4227.859, 7675.456)	− 0.537 (− 0.592, − 0.485)	9.879 (8.041, 12.308)	− 0.252 (− 0.337, − 0.184)	73.552 (54.890, 93.474)	− 0.371 (− 0.412, − 0.318)	0	0
GBD regions								
Andean Latin America	0	0	2.142 (1.720, 2.700)	− 0.091 (− 0.130, − 0.051)	187.418 (142.285, 235.293)	− 0.330 (− 0.377, − 0.283)	2606.068 (2127.284, 3170.801)	− 0.271 (− 0.358, − 0.184)
Australasia	0	0	0.956 (0.735, 1.247)	− 0.010 (− 0.045, 0.025)	0	0	0	0
Caribbean	2567.385 (1709.770, 3683.908)	0.163 (0.096, 0.218)	0.344 (0.264, 0.444)	− 0.289 (− 0.324, − 0.254)	105.260 (76.076, 134.154)	− 0.125 (− 0.169, − 0.081)	0	0
Central Asia	0	0	23.040 (18.720, 28.196)	0.072 (− 0.023, 0.174)	28.375 (19.262, 40.765)	− 0.357 (− 0.409, − 0.297)	3.778 (3.314, 4.338)	− 0.911 (− 0.924, − 0.894)
Central Europe	0	0	10.760 (9.047, 12.770)	0.041 (− 0.009, 0.074)	81.765 (56.284, 110.104)	− 0.200 (− 0.252, − 0.131)	0	0
Central Latin America	280.794 (192.045, 399.069)	− 0.183 (− 0.366, 0.006)	1.982 (1.495, 2.646)	− 0.088 (− 0.124, − 0.053)	216.418 (160.269, 271.397)	− 0.171 (− 0.206, − 0.136)	0	0
Central sub-Saharan Africa	7947.466 (5702.598, 11,151.884)	− 0.584 (− 0.638, − 0.505)	2.952 (2.355, 3.721)	− 0.266 (− 0.363, − 0.182)	191.352 (144.103, 234.995)	− 0.287 (− 0.332, − 0.241)	0	0
East Asia	733.949 (559.287, 955.907)	− 0.400 (− 0.453, − 0.354)	6.598 (5.000, 8.660)	1.536 (− 1.339, 1.765)	35.014 (24.205, 48.692)	− 0.373 (− 0.417, − 0.305)	1862.448 (1640.720, 2161.612)	− 0.416 (− 0.456, − 0.371)
Eastern Europe	0	0	15.090 (12.203, 18.264)	− 0.145 (− 0.160, − 0.128)	61.032 (43.687, 80.572)	− 0.331 (− 0.393, − 0.266)	685.271 (601.848, 780.557)	0.004 (− 0.022, 0.030)
Eastern sub-Saharan Africa	11,617.619 (8557.990, 15,093.062)	− 0.424 (− 0.484, − 0.376)	17.242 (13.825, 21.724)	− 0.338 (− 0.399, − 0.285)	83.317 (61.144, 110.274)	− 0.355 (− 0.413, − 0.292)	0	0
High-income Asia Pacific	0	0	0.685 (0.504, 0.940)	0.065 (− 0.022, 0.115)	16.115 (10.893, 22.908)	− 0.026 (− 0.170, 0.165)	821.489 (731.265, 915.189)	0.110 (0.010, 0.219)
High-income North America	0	0	0.898 (0.654, 1.233)	− 0.104 (− 0.133, − 0.072)	81.847 (57.544, 108.681)	0.155 (0.067, 0.251)	0	0
North Africa and Middle East	1127.855 (805.087, 1597.262)	− 0.833 (− 0.876, − 0.776)	15.243 (12.529, 18.653)	− 0.227 (− 0.341, − 0.179)	0.503 (0.336, 0.748)	− 0.519 (− 0.561, − 0.478)	135.784 (106.494, 171.024)	− 0.357 (− 0.454, − 0.245)
Oceania	0	0	0.131 (0.086, 0.197)	0.033 (− 0.079, 0.161)	54.963 (37.607, 76.663)	− 0.336 (− 0.385, − 0.277)	0	0
South Asia	0	0	10.933 (8.808, 13.602)	0.108 (− 0.049, 0.160)	51.574 (38.059, 65.688)	− 0.424 (− 0.491, − 0.340)	0	0
Southeast Asia	142.812 (97.279, 244.126)	− 0.599 (− 0.642, − 0.545)	0.848 (0.662, 1.106)	− 0.052 (− 0.072, − 0.030)	21.673 (15.271, 30.209)	− 0.234 (− 0.282, − 0.166)	652.032 (593.448, 711.677)	− 0.750 (− 0.768, − 0.727)
Southern Latin America	0	0	38.151 (32.047, 45.242)	− 0.433 (− 0.489, − 0.361)	80.414 (56.756, 106.690)	− 0.179 (− 0.244, − 0.098)	0	0
Southern sub-Saharan Africa	5950.951 (4379.207, 7964.835)	− 0.229 (− 0.283, − 0.180)	1.022 (0.797, 1.334)	− 0.109 (− 0.138, − 0.085)	208.321 (155.272, 258.385)	− 0.169 (− 0.222, − 0.113)	0	0
Tropical Latin America	2395.445 (1751.943, 3105.576)	− 0.140 (− 0.206, − 0.063)	1.448 (1.130, 1.837)	− 0.111 (− 0.128, − 0.098)	122.025 (88.686, 159.660)	− 0.278 (− 0.332, − 0.219)	0	0
Western Europe	0	0	2.354 (1.969, 2.852)	− 0.258 (− 0.292, − 0.219)	10.511 (7.499, 14.209)	− 0.030 (− 0.103, 0.062)	18.185 (13.819, 23.228)	− 0.474 (− 0.562, − 0.385)
Western sub-Saharan Africa	13,433.552 (9651.799, 17,812.075)	− 0.421 (− 0.468, − 0.377)	1.408 (1.104, 1.840)	− 0.308 (− 0.541, − 0.185)	68.981 (50.143, 91.169)	− 0.258 (− 0.293, − 0.218)	0	0

*ASPRs* Age-standardized prevalence rates, *EAPCs* estimated annual percentage changes, *SDI* Socio-demographic Index, *UI* uncertainty intervals, *CI* confidence interval

#### The prevalence burden of cystic echinococcosis

In 2021, there were approximately 633,404 cystic echinococcosis cases (95% UI: 517,477, 782,469) globally, with an ASPR of 7.691 cases (95% UI: 6.269, 9.507) per 100,000 population. From 1990 to 2021, the EAPC of ASPR was 0.044 (95% *CI*: − 0.025, 0.100), showing no obvious change (Table 1). Compared to the other four SDI regions in 2021, the high SDI region had significantly lower case numbers and ASPR (Table S1). Among the GBD regions, the highest number of cases was recorded in South Asia (199,794, 95% UI: 161,370, 248,813), followed by East Asia (114,589, 95% UI: 86,308, 147,199), and North Africa and the Middle East (94,754, 95% UI: 77,603, 117,793). The region with the highest ASPRs was Southern Latin America (38.151 per 100,000 population, 95% UI: 32.047, 45.242), followed by Central Asia (23.040, 95% UI: 18.720, 28.196), and Eastern sub-Saharan Africa (17.242, 95% UI: 13.825, 21.724). Additionally, six out of the 21 GBD regions showed increasing trends in ASPR from 1990 to 2021, with East Asia experiencing the highest annual change (1.536, 95% *CI*: − 1.339, 1.765).

#### The prevalence burden of cysticercosis

In 2021, there were an estimated 4.357 million cases of cysticercosis worldwide (95% UI: 3.150, 5.716 million), with an ASPR of 51.269 (95% UI: 37.166, 67.279) cases per 100,000 population (Table S1, Table 1). Among the five SDI regions, the middle SDI region had the highest number of cases, with 1.591 million (95% UI: 1.143, 2.091 million). The low SDI region reported the highest ASPR, with 73.552 (95% UI: 54.890, 93.474) cases per 100,000 population. While the number of cases in all five SDI regions increased over time, the ASPR declined in four regions (excluding the high SDI region), with the high-middle SDI region experiencing the largest decrease (the EAPC is − 0.376, 95% *CI*: − 0.418, − 0.319). Notably, the low SDI region’s EAPC was − 0.371 (95% *CI*: − 0.412, − 0.318), closely mirrored that of the high-middle SDI region (Table 1, Fig S1). By GBD region analysis, the highest number of cysticercosis cases in 2021 was observed in South Asia (755,683 cases, 95% UI: 551,993, 972,079), followed by East Asia (725,883 cases, 95% UI: 494,719, 1,018,980), and Central Latin America (562,969 cases, 95% UI: 410,577, 708,799) (Table S1). Central Latin America reported the highest ASPR at 216.418 cases per 100,000 population (95% UI: 160.269, 271.397), followed by Southern sub-Saharan Africa (208.321, 95% UI: 155.272, 258.385) and Central sub-Saharan Africa (191.352, 95% UI: 144.103, 234.995). From 1990 to 2021, the ASPR of cysticercosis declined across all GBD regions except the high-income North America, while the most significant decrease was observed in North Africa and the Middle East with the EAPC of − 0.519 (95% *CI*: − 0.561, − 0.478).

#### The prevalence burden of FBTs

For FBTs, there were an estimated 44.466 million cases worldwide (95% UI: 40.017, 50.034 million) (Table S1), with an ASPR of 526.740 (95% UI: 473.703, 593.254) cases per 100,000 population in 2021 (Table 1). Globally, the EAPC of ASPR was − 0.501 (95% *CI*: − 0.527, − 0.469) from 1990 to 2021 (Table 1). Among the SDI regions, no case was reported in the low SDI region. The middle SDI region had the highest number of cases, and the high-middle SDI region had the highest ASPR (1232.730 cases per 100,000 population, 95% UI: 1094.886, 1404.875). Generally, all SDI regions experienced a declining trend in ASPR from 1990 to 2021 (Table 1, Fig. S1). Eight GBD regions reported cases of FBTs in 2021, and the region with the highest number of cases was East Asia (33.317 million, 95% UI: 29.251, 38.354 million), significantly surpassing the cases reported in other GBD regions (Table S1). Andean Latin America showed the highest ASPR (2606.068 cases per 100,000 population, 95% UI: 2127.284, 3170.801), followed by East Asia (1862.448, 95% UI: 1640.720, 2161.612). From 1990 to 2021, an increase in ASPR was observed in high-income Asia Pacific (the EAPC = 0.110, 95% *CI*: 0.010, 0.219), but other regions exhibited a declining trend during this period. Central Asia, which recorded the lowest ASPR, also experienced the steepest decline of the EAPC at − 0.911 (95% *CI*: − 0.924, − 0.894), followed by Southeast Asia (− 0.750, 95% *CI*: − 0.768, − 0.727).

### The mortality and temporal trend of zIDPs

In 2021, there were an estimated 12,858 deaths due to schistosomiasis (95% UI: 11,335, 14,388). The ASMR was 0.153 (95% UI: 0.135, 0.172) deaths per 100,000 population, with an EAPC of − 0.667 (95% *CI*: − 0.705, − 0.625) from 1990 to 2021 (Fig. S2). The low SDI region had the highest number of deaths (8430, 95% UI: 7265, 9681) and the highest ASMR (1.369 deaths per 100,000 population, 95% UI: 1.197, 1.562). Among the 10 GBD regions reported schistosomiasis cases, Western, Eastern, and Central sub-Saharan Africa had the highest numbers of deaths and ASMRs (Table 2, Table S2). All SDI and GBD regions showed significant declines in mortality rates from 1990 to 2021 (Table 2, Fig. S2).

**Table 2:** Age-standardized mortality rate of zoonotic infectious diseases of poverty in 1990 and 2021, and estimated annual changes from 1990 to 2021

Regional classification	Schistosomiasis		Cystic echinococcosis		Cysticercosis	
	ASRs per 100,000, 2021 (95% UI)	EAPCs, 1990 to 2021 (95% *CI*)	ASRs per 100,000, 2021 (95% UI)	EAPCs, 1990 to 2021 (95% *CI*)	ASRs per 100,000, 2021 (95% UI)	EAPCs, 1990 to 2021 (95% *CI*)
Global	0.153 (0.135, 0.172)	− 0.667 (− 0.705, − 0.625)	0.017 (0.012, 0.022)	− 0.774 (− 0.839, − 0.691)	0.020 (0.014, 0.028)	− 0.508 (− 0.662, − 0.280)
SDI regions						
High SDI	0.003 (0.002, 0.003)	− 0.308 (− 0.535, 0.062)	0.001 (0.001, 0.002)	− 0.750 (− 0.859, − 0.559)	0.000 (0.000, 0.000)	− 0.727 (− 0.853, − 0.495)
High-middle SDI	0.010 (0.008, 0.012)	− 0.696 (− 0.757, − 0.610)	0.009 (0.007, 0.011)	− 0.807 (− 0.868, − 0.727)	0.001 (0.001, 0.001)	− 0.840 (− 0.906, − 0.725)
Middle SDI	0.050 (0.045, 0.055)	− 0.844 (− 0.863, − 0.820)	0.011 (0.008, 0.014)	− 0.787 (− 0.857, − 0.695)	0.009 (0.006, 0.012)	− 0.716 (− 0.831, − 0.551)
Low-middle SDI	0.187 (0.163, 0.210)	− 0.657 (− 0.696, − 0.615)	0.031 (0.022, 0.041)	− 0.763 (− 0.833, − 0.678)	0.023 (0.015, 0.031)	− 0.573 (− 0.736, − 0.273)
Low SDI	1.369 (1.197, 1.562)	− 0.699 (− 0.740, − 0.654)	0.051 (0.035, 0.068)	− 0.784 (− 0.855, − 0.692)	0.123 (0.082, 0.184)	− 0.561 (− 0.688, − 0.367)
GBD regions						
Andean Latin America	0	0	0.004 (0.002, 0.006)	− 0.821 (− 0.911, − 0.664)	0.042 (0.027, 0.061)	− 0.749 (− 0.851, − 0.596)
Australasia	0	0	0.002 (0.001, 0.002)	− 0.805 (− 0.907, − 0.619)	0	0
Caribbean	0.069 (0.050, 0.091)	− 0.491 (− 0.651, − 0.257)	0.001 (0.000, 0.001)	− 0.760 (− 0.880, − 0.511)	0.084 (0.057, 0.114)	− 0.443 (− 0.641, − 0.125)
Central Asia	0	0	0.013 (0.008, 0.019)	− 0.712 (− 0.845, − 0.499)	0.000 (0.000, 0.001)	− 0.688 (− 0.851, − 0.345)
Central Europe	0	0	0.008 (0.004, 0.011)	− 0.792 (− 0.891, − 0.629)	0.000 (0.000, 0.000)	− 0.766 (− 0.889, − 0.492)
Central Latin America	0.020 (0.014, 0.027)	− 0.553 (− 0.712, − 0.331)	0.001 (0.001, 0.001)	− 0.752 (− 0.876, − 0.530)	0.047 (0.030, 0.065)	− 0.679 (− 0.816, − 0.449)
Central sub-Saharan Africa	3.021 (2.365, 3.721)	− 0.597 (− 0.696, − 0.464)	0.033 (0.021, 0.047)	− 0.766 (− 0.858, − 0.639)	0.190 (0.139, 0.247)	− 0.525 (− 0.660, − 0.339)
East Asia	0.026 (0.021, 0.031)	− 0.911 (− 0.930, − 0.885)	0.004 (0.003, 0.005)	− 0.867 (− 0.894, − 0.831)	0.002 (0.001, 0.002)	− 0.882 (− 0.942, − 0.778)
Eastern Europe	0	0	0.010 (0.005, 0.014)	− 0.722 (− 0.858, − 0.451)	0.001 (0.000, 0.001)	− 0.530 (− 0.785, 0.047)
Eastern sub-Saharan Africa	2.049 (1.819, 2.330)	− 0.753 (− 0.795, − 0.707)	0.068 (0.044, 0.092)	− 0.803 (− 0.875, − 0.701)	0.218 (0.144, 0.309)	− 0.579 (− 0.705, − 0.404)
High-income Asia Pacific	0	0	0.000 (0.000, 0.000)	− 0.865 (− 0.935, − 0.740)	0.000 (0.000, 0.000)	− 0.901 (− 0.952, − 0.800)
High-income North America	0	0	0.001 (0.000, 0.001)	− 0.686 (− 0.845, − 0.389)	0.001 (0.000, 0.001)	− 0.633 (− 0.822, − 0.233)
North Africa and Middle East	0.146 (0.120, 0.175)	− 0.814 (− 0.846, − 0.775)	0.059 (0.043, 0.076)	− 0.794 (− 0.853, − 0.720)	0.000 (0.000, 0.001)	− 0.824 (− 0.915, − 0.637)
Oceania	0	0	0.005 (0.003, 0.008)	− 0.680 (− 0.840, − 0.370)	0.014 (0.008, 0.022)	− 0.451 (− 0.734, − 0.164)
South Asia	0	0	0.031 (0.022, 0.041)	− 0.764 (− 0.837, − 0.666)	0.004 (0.002, 0.006)	− 0.801 (− 0.892, − 0.637)
Southeast Asia	0.040 (0.034, 0.046)	− 0.366 (− 0.476, − 0.235)	0.012 (0.007, 0.016)	− 0.768 (− 0.871, − 0.624)	0.002 (0.001, 0.003)	− 0.783 (− 0.891, − 0.562)
Southern Latin America	0	0	0.010 (0.006, 0.014)	− 0.778 (− 0.876, − 0.621)	0.001 (0.001, 0.002)	− 0.881 (− 0.941, − 0.764)
Southern sub-Saharan Africa	0.565 (0.467, 0.677)	− 0.177 (− 0.329, 0.019)	0.018 (0.011, 0.026)	− 0.644 (− 0.811, − 0.335)	0.102 (0.065, 0.143)	− 0.309 (− 0.603–0.300)
Tropical Latin America	0.203 (0.184, 0.222)	− 0.734 (− 0.762, − 0.704)	0.003 (0.002, 0.004)	− 0.765 (− 0.879, − 0.563)	0.044 (0.028, 0.061)	− 0.651 (− 0.799, − 0.379)
Western Europe	0	0	0.002 (0.001, 0.003)	− 0.801 (− 0.897, − 0.646)	0.000 (0.000, 0.000)	− 0.808 (− 0.905, − 0.607)
Western sub-Saharan Africa	1.740 (1.486, 2.016)	− 0.637 (− 0.690, − 0.572)	0.023 (0.013, 0.034)	− 0.773 (− 0.862, − 0.633)	0.107 (0.059, 0.179)	− 0.557 (− 0.745, − 0.272)

*ASMRs* Age-standardized mortality rates, *EAPCs* estimated annual percentage changes, *SDI* Socio-demographic Index, *UI* uncertainty intervals; *CI* confidence interval

In 2021, the estimated global deaths and ASMR of cystic echinococcosis were 1364 (95% UI: 986, 1775) and 0.017 (95% UI: 0.012, 0.022) deaths per 100,000 population, respectively. The low-middle SDI region recorded the highest number of deaths (492, 95% UI: 361, 631), followed by the low SDI region with 411 deaths (95% UI: 275, 563). Among the 21 GBD regions, South Asia had the highest death count with 491 deaths (95% UI: 350, 644) and Eastern sub-Saharan Africa recorded the highest ASMR (0.068, 95% UI: 0.044, 0.092) deaths per 100,000 population in 2021.The EAPC of ASMR was − 0.774 (95% *CI*: − 0.839, − 0.691) globally, showing a significant decrease over the past three decades. This trend was observed in all SDI and GBD regions (Table 2, Fig. S2).

For cysticercosis, the global death toll in 2021 was 1643 (95% UI: 1114, 2287), with an ASMR of 0.020 deaths per 100,000 population (95% UI: 0.014, 0.028). The majority of deaths occurred in the low and low-middle SDI regions, as well as in sub-Saharan Africa (Table S2). Similarly to CE, the ASMR of cysticercosis showed a declining trend globally with an EAPC of − 0.508 (95% *CI*: − 0.662, − 0.280) from 1990 to 2021 (Table 2, Fig. S2). There was no data reported on the deaths caused by FBTs from the GBD 2021 study.

### The DALYs and temporal trend of zIDPs

In 2021, the DALYs caused by schistosomiasis were 1,746,333 (95% UI: 1,038,122, 2,984,204), with an age-standardized DALY rate of 21.895 (95% UI: 12.937, 37.278) per 100,000 population. From 1990 to 2021, an EAPC of − 0.414 (95% *CI*: − 0.520, − 0.319) was observed, showing a significant global decline in the age-standardized DALY rate for schistosomiasis. By SDI analysis, the low SDI region reported the highest burden of schistosomiasis with the highest DALYs and age-standardized DALY rate at 94.479 (95% UI: 65.769, 146.991) per 100,000 population, although significant decline in the age-standardized DALY rate observed in this region with an EAPC of − 0.641 (95% *CI*: − 0.692, − 0.596) (Fig. S3). In the 10 GBD regions where schistosomiasis cases were recorded, Central, Eastern, and Western sub-Saharan Africa had the highest DALYs and age-standardized DALY rates (Table S3). All schistosomiasis-endemic GBD regions exhibited a decrease in the age-standardized DALY rate, with the most significant decline observed in North Africa and the Middle East according to the EAPC (− 0.817, 95% *CI*: − 0.847, − 0.779) (Table 3).

**Table 3:** Age-standardized DALY rate of zoonotic infectious diseases of poverty in 1990 and 2021, and estimated annual changes from 1990 to 2021

Regional classification	Schistosomiasis		Cystic echinococcosis		Cysticercosis		Food-borne trematodiases	
	Age-standardized DALY rates per 100,000, 2021 (95% UI)	EAPCs, 1990 to 2021 (95% *CI*)	Age-standardized DALY rates per 100,000, 2021 (95% UI)	EAPCs, 1990 to 2021 (95% *CI*)	Age-standardized DALY rates per 100,000, 2021 (95% UI)	EAPCs, 1990 to 2021 (95% *CI*)	Age-standardized DALY rate per 100,000, 2021 (95% UI)	EAPCs, 1990 to 2021 (95% *CI*)
Global	21.895 (12.937, 37.278)	− 0.414 (− 0.520, 0.319)	1.320 (0.991, 1.688)	− 0.718 (− 0.792, − 0.622)	14.622 (9.332, 21.343)	− 0.385 (− 0.470, − 0.286)	11.788 (6.727, 19.461)	− 0.542 (− 0.595, − 0.446)
SDI regions								
High SDI	0.486 (0.230, 0.991)	0.359 (− 0.104, 0.927)	0.172 (0.118, 0.235)	− 0.443 (− 0.611, − 0.224)	7.766 (4.286, 12.480)	− 0.105 (− 0.286, 0.076)	4.479 (2.804, 6.736)	− 0.125 (− 0.230, 0.037)
High-middle SDI	1.199 (0.659, 2.345)	− 0.540 (− 0.638, − 0.449)	0.992 (0.711, 1.314)	− 0.618 (− 0.707, − 0.518)	8.909 (5.128, 14.473)	− 0.492 (− 0.599, − 0.376)	28.026 (15.418, 48.730)	− 0.310 (− 0.403, − 0.107)
Middle SDI	12.104 (6.100, 24.150)	− 0.449 (− 0.593, − 0.323)	0.919 (0.656, 1.183)	− 0.649 (− 0.749, − 0.529)	15.939 (9.979, 23.303)	− 0.416 (− 0.522, − 0.293)	16.636 (9.321, 27.683)	− 0.667 (− 0.702, − 0.604)
Low-middle SDI	26.807 (15.394, 48.050)	− 0.443 (− 0.527, − 0.359)	1.761 (1.354, 2.208)	− 0.759 (− 0.817, − 0.677)	19.377 (12.783, 27.350)	− 0.482 (− 0.557, − 0.385)	1.669 (0.669, 3.033)	− 0.477 (− 0.645, − 0.362)
Low SDI	94.479 (65.769, 146.991)	− 0.641 (− 0.692, − 0.596)	2.891 (2.055, 3.806)	− 0.799 (− 0.857, − 0.722)	29.222 (20.420, 40.536)	− 0.440 (− 0.522, − 0.331)	0	0
GBD regions								
Andean Latin America	0	0	0.303 (0.208, 0.422)	− 0.752 (− 0.852, − 0.576)	51.116 (29.133, 82.349)	− 0.500 (− 0.699, − 0.236)	56.058 (19.031, 106.095)	− 0.300 (− 0.452, − 0.168)
Australasia	0	0	0.120 (0.083, 0.158)	− 0.652 (− 0.770, − 0.452)	0	0	0	0
Caribbean	20.278 (9.596, 43.342)	− 0.035 (− 0.185, 0.071)	0.054 (0.035, 0.077)	− 0.665 (− 0.819, − 0.381)	35.819 (22.778, 51.604)	− 0.245 (− 0.412, − 0.055)	0	0
Central Asia	0	0	2.187 (1.494, 3.015)	− 0.371 (− 0.544, − 0.169)	7.392 (4.012, 12.557)	− 0.428 (− 0.619, − 0.191)	0.090(0.043, 0.143)	− 0.912 (− 0.930, − 0.887)
Central Europe	0	0	1.001 (0.717, 1.363)	− 0.501 (− 0.638, − 0.330)	18.452 (9.999, 29.947)	− 0.340 (− 0.519, − 0.131)	0	0
Central Latin America	2.428 (1.307, 4.701)	− 0.335 (− 0.497, − 0.175)	0.186 (0.124, 0.257)	− 0.461 (− 0.620, − 0.269)	60.291 (37.933, 87.265)	− 0.333 (− 0.449, − 0.193)	0	0
Central sub-Saharan Africa	161.892 (116.491, 234.202)	− 0.603 (− 0.671, − 0.519)	1.500 (1.006, 2.033)	− 0.816 (− 0.876, − 0.730)	71.991 (46.402, 103.653)	− 0.393 (− 0.550, − 0.218)	0	0
East Asia	5.348 (2.553, 10.630)	− 0.679 (− 0.783, − 0.581)	0.602 (0.398, 0.823)	− 0.571 (− 0.705, − 0.416)	8.529 (4.971, 13.690)	− 0.542 (− 0.668, − 0.400)	42.611 (21.295, 77.043)	− 0.474 (− 0.547, − 0.289)
Eastern Europe	0	0	1.399 (0.973, 1.902)	− 0.440 (− 0.581, − 0.260)	14.286 (7.858, 22.819)	− 0.410 (− 0.553, − 0.248)	15.611 (7.926, 24.699)	0.005 (− 0.040, 0.048)
Eastern sub-Saharan Africa	157.580 (103.564, 261.725)	− 0.648 (− 0.716, − 0.573)	4.150 (3.017, 5.359)	− 0.804 (− 0.864, − 0.717)	36.364 (25.530, 50.939)	− 0.488 (− 0.570, − 0.391)	0	0
High-income Asia Pacific	0	0	0.057 (0.036, 0.083)	− 0.488 (− 0.653, − 0.294)	3.356 (1.229, 7.175)	− 0.281 (− 0.690, 0.603)	14.270 (9.179, 20.240)	0.170 (0.042, 0.319)
High-income North America	0	0	0.092 (0.062, 0.128)	− 0.407 (− 0.566, − 0.217)	16.824 (9.273, 26.863)	0.057 (− 0.151, 0.308)	0	0
North Africa and Middle East	13.859 (8.460, 24.214)	− 0.817 (− 0.847, − 0.779)	2.967 (2.303, 3.724)	− 0.784 (− 0.834, − 0.714)	0.166 (0.097, 0.281)	− 0.646 (− 0.741, − 0.518)	2.758 (0.724, 5.473)	− 0.359 (− 0.467, − 0.239)
Oceania	0	0	0.208 (0.124, 0.317)	− 0.670 (− 0.831, − 0.370)	19.007 (11.113, 30.174)	− 0.369 (− 0.546, − 0.135)	0	0
South Asia	0	0	1.923 (1.470, 2.423)	− 0.741 (− 0.804, − 0.654)	14.756 (9.489, 21.409)	− 0.545 (− 0.630, − 0.430)	0	0
Southeast Asia	2.444 (1.742, 3.777)	− 0.500 (− 0.551, − 0.430)	0.474 (0.304, 0.653)	− 0.796 (− 0.880, − 0.656)	6.234 (3.629, 9.930)	− 0.371 (− 0.512, − 0.212)	13.103 (8.160, 19.119)	− 0.774 (− 0.804, − 0.720)
Southern Latin America	0	0	2.994 (2.021, 4.194)	− 0.525 (− 0.616, − 0.422)	19.273 (9.676, 32.765)	− 0.328 (− 0.583–0.054)	0	0
Southern sub-Saharan Africa	68.756 (41.605, 120.936)	− 0.235 (− 0.298, − 0.171)	0.806 (0.493, 1.175)	− 0.670 (− 0.833, − 0.354)	66.809 (42.669, 94.280)	− 0.252 (− 0.381, − 0.100)	0	0
Tropical Latin America	22.636 (13.226, 39.487)	− 0.479 (− 0.595, − 0.363)	0.214 (0.146, 0.289)	− 0.656 (− 0.780, − 0.441)	33.922 (21.634, 51.469)	− 0.419 (− 0.548, − 0.283)	0	0
Western Europe	0	0	0.233 (0.165, 0.321)	− 0.619 (− 0.719, − 0.479)	2.108 (0.911, 3.970)	− 0.145 (− 0.539–0.537)	0.373 (0.101, 0.748)	− 0.473 (− 0.571, − 0.377)
Western sub-Saharan Africa	171.127 (106.537, 289.245)	− 0.524 (− 0.588, − 0.465)	1.271 (0.723, 1.937)	− 0.810 (− 0.888, − 0.685)	26.712 (17.834, 39.022)	− 0.397 (− 0.506, − 0.295)	0	0

*EAPCs* estimated annual percentage changes, *SDI* Socio-demographic Index, *UI* uncertainty intervals; *CI* confidence interval

It was estimated that cystic echinococcosis caused 105,071 DALYs (95% UI: 78,967, 133,309) and the age-standardized DALY rate of cystic echinococcosis was 1.320 (95% UI: 0.991, 1.688) per 100,000 population in 2021. From 1990 to 2021, the age-standardized DALY rate recorded a clear decrease with an EAPC at − 0.718 (95% *CI*: − 0.792, − 0.622) (Table 3, Fig. S3). The highest number of DALYs occurred in the low-middle and low SDI regions, and the low SDI region recorded the highest age-standardized DALY rate (2.891, 95% UI: 2.055, 3.806). Among the GBD regions, the largest DALYs burden was found in South Asia (34,006 DALYs, 95% UI: 25,804, 43,190), followed by Eastern sub-Saharan Africa (17,337 DALYs, 95% UI: 12,042, 23,167), and North Africa and the Middle East (17,319 DALYs, 95% UI: 13,165, 21,974). The highest age-standardized DALY rates were observed in Eastern sub-Saharan Africa (4.150 per 100,000 population, 95% UI: 3.017, 5.359), Southern Latin America (2.994, 95% UI: 2.021, 4.194), and North Africa and the Middle East (2.976, 95% UI: 2.303, 3.724). All GBD regions experienced a significant decrease in age-standardized DALY rates, with Western sub-Saharan Africa showing the steepest decline (EAPC: − 0.810, 95% *CI*: − 0.888, − 0.685).

For cysticercosis, the estimated number of DALYs was 1.236 million (95% UI: 0.78, 1.808 million), and the age-standardized DALY rate was 14.622 (95% UI: 9.332 to 21.343) per 100,000 population in 2021. From 1990 to 2021, the global age-standardized DALY rate showed an average annual decrease of − 0.385 (95% UI: − 0.470, − 0.286) (Table 3, Fig. S3). Among the five SDI regions, the low and low-middle regions ranked the first and second places with high age-standardized DALY rates (Table 3). The highest age-standardized DALY rates were observed in Central sub-Saharan Africa (71.991 per 100,000 population, 95% UI: 46.402, 103.653), followed by Southern sub-Saharan Africa (66.809, 95% UI: 42.669, 94.280) and Central Latin America (60.291, 95% UI: 37.933, 87.265). All SDI regions and GBD regions, except for High-income North America, experienced declines in the age-standardized DALY rate for cysticercosis. The most significant decrease was noted in North Africa and the Middle East according to the EAPC (− 0.646, 95% *CI*: − 0.741, − 0.518).

In 2021, FBTs resulted in an estimated 998,028 DALYs (95% UI: 569,766, 1,638,112), with an age-standardized DALY rate of 11.788 (95% UI: 6.727, 19.461) per 100,000 population. The EAPC of the age-standardized DALY rate was − 0.542 (95% *CI*: − 0.595, − 0.446, Table 3), presenting a significant decline trend from 1990 to 2021 (Fig. S3). Among the four SDI regions that reported cases, the middle and high-middle SDI regions recorded the highest age-standardized DALY rates (Table 3, Table S3). In the eight GBD regions with reported cases, East Asia had the largest number of DALYs at 768,297 (95% UI: 383,883, 1,367,826), while Andean Latin America had the highest age-standardized DALY rate at 56.058 per 100,000 population (95% UI: 19.031, 106.095). An increase in the age-standardized DALY rate was observed in High-income Asia Pacific (EAPC = 0.170, 95% *CI*: 0.042, 0.319), whereas all other regions exhibited a downward trend (Table 3).

### The disease burden of zIDPs by age and gender

**Fig. 1:**
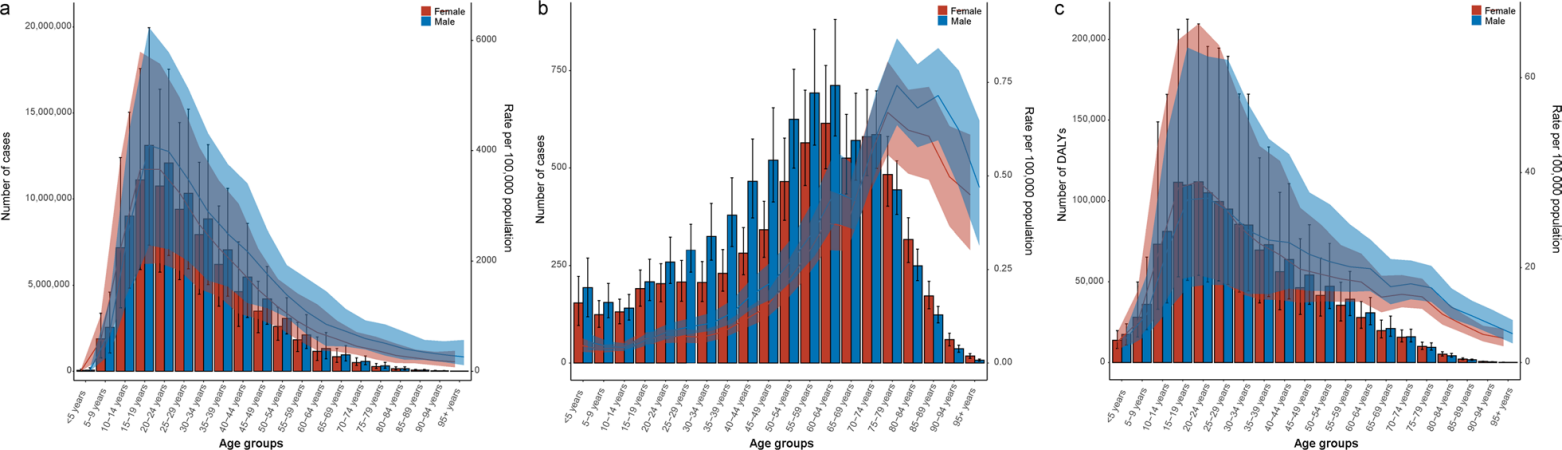
The prevalence (**a**), deaths (**b**), and DALYs (**c**) of schistosomiasis for both gender and all age groups. DALYs: disability-adjusted life years. The left axis was the cases (**a**), deaths (**b**), and number of DALYs (**c**), and the right axis was the age-standardized rates of prevalence (**a**), mortality (**b**), and DALYs (**c**). The fold lines represent the change of the age-standardized rates (per 100,000 population) in different age groups. The shaded areas represent 95% uncertainty interval (UI) of the fold lines

Analyzed by age groups, the majority of schistosomiasis cases occurred in groups aged from 10 to 49, while the 10–29 age groups had the highest number of cases. The ASPR increased up until the 15–19 age group, and then decreased with age (Fig. 1a). Mortality due to schistosomiasis was most prevalent in individuals aged 35 to 84 years and peaked in the 60–64 age group. The ASMR was similar for both males and females, showing an upward trend and peaked in the 75–79 age group (Fig. 1b). The burden of DALYs due to schistosomiasis was concentrated in the 10–44 age groups, with the 15–19 and 20–24 age groups bearing the highest burden. There was no significant difference in the ASPR, ASMR and age-standardized DALYs rate between males and females (Fig. 1c).

**Fig. 2:**
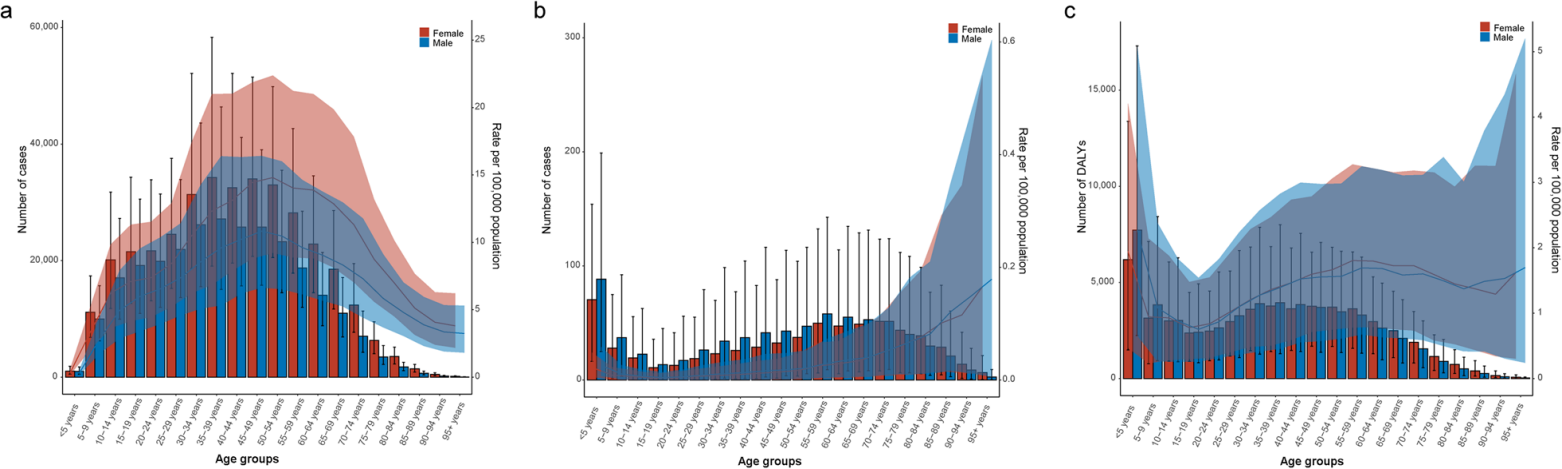
The prevalence (**a**), deaths (**b**), and DALYs (**c**) of cystic echinococcosis for both gender and all age groups. DALYs: disability-adjusted life years. The left axis was the new cases (**a**), cases (**b**), deaths (**c**), and number of DALYs (**d**), and the right axis was the age-standardized rates of incidence (**a**), prevalence (**b**), mortality (**c**), and DALYs (**c**). The fold lines represent the change of the age-standardized rates (per 100,000 population) in different age groups. The shaded areas represent 95% uncertainty interval (UI) of the fold lines

For cystic echinococcosis, cases were concentrated in the population of 5–69 years old, particularly in the population aged from 30 to 54. The ASPR increased with age and peaked in the 45–49 age group for males and 50–54 age group for females, followed by declined (Fig. 2a). The highest mortality of cystic echinococcosis observed in group with age less than 5 and the highest ASMR occurred in those over 95 years old (Fig. 2b). The highest number of DALYs and the highest age-standardized DALYs rate were found in the group less than 5 years old. There was also no significant difference between males and females in ASPR, ASMR and the age-standardized DALYs rate due to cystic echinococcosis (Fig. 2c).

**Fig. 3:**
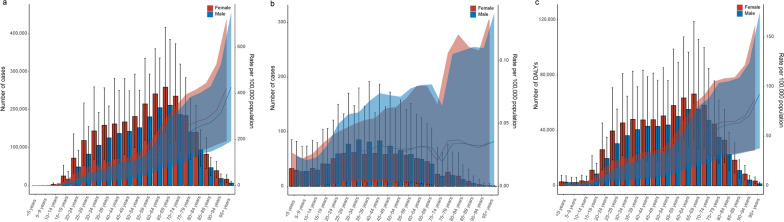
The prevalence (**a**), deaths (**b**), and DALYs (**c**) of cysticercosis for both gender and all age groups. DALYs: disability-adjusted life years. The left axis was the cases (**a**), deaths (**b**), and number of DALYs (**c**), and the right axis was the age-standardized rates of prevalence (**a**), mortality (**b**), and DALYs (**c**). The fold lines represent the change of the age-standardized rates (per 100,000 population) in different age groups. The shaded areas represent 95% uncertainty interval (UI) of the fold lines

For cysticercosis, most cases and DALYs were concentrated in the age groups between 24 and 89 years. The ASPR, ASMR and the age-standardized DALYs rate for both genders increased with age, while the ASPR and ASMR reached the peak values in the over-95 age group (Fig. 3a–c). Notably, there was no significant difference in the ASPR, ASMR, and age-standardized DALYs rate between males and females for cysticercosis.

**Fig. 4:**
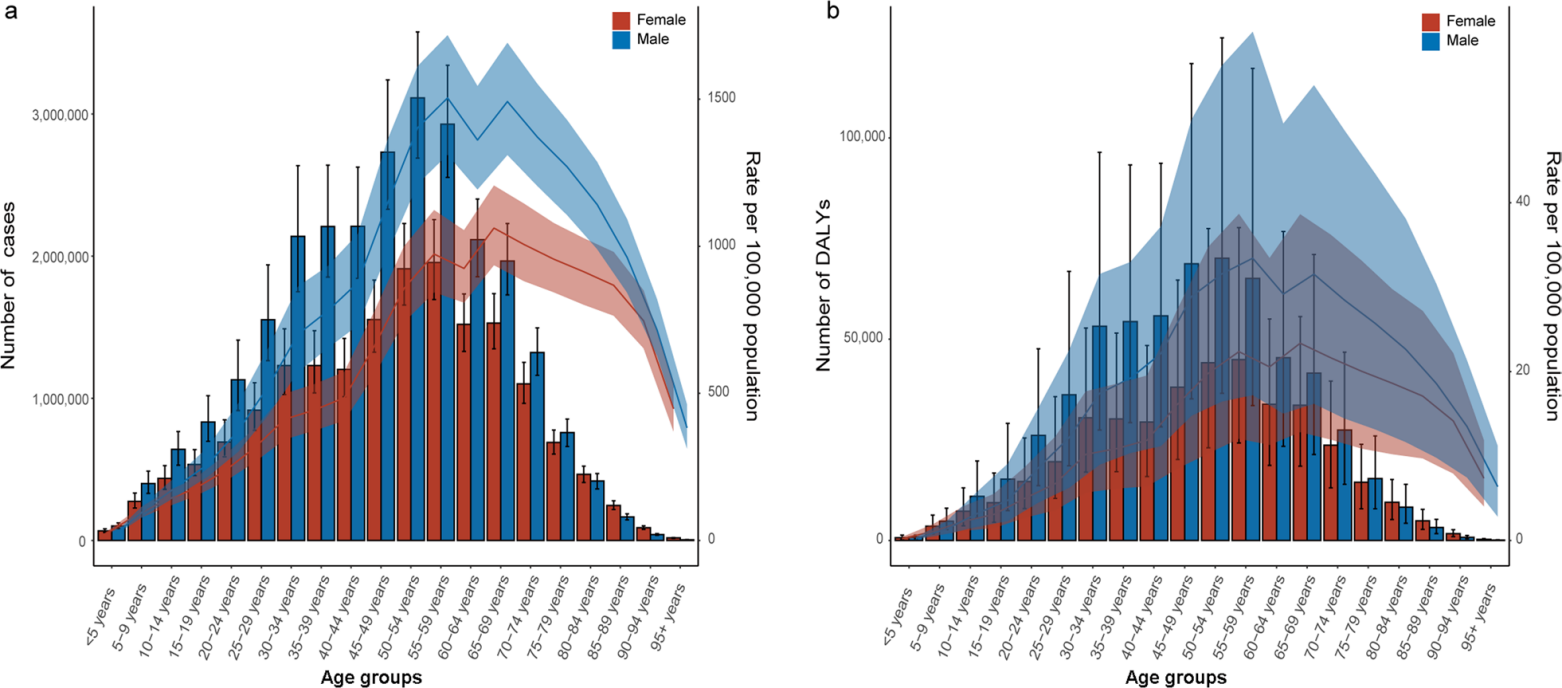
The prevalence (**a**), and DALYs (**b**) of foodborne trematodiases for both gender and all age groups. DALYs: disability-adjusted life years. The left axis was the cases (**a**), and number of DALYs (**b**), and the right axis was the age-standardized rates of prevalence (**a**), and DALYs (**b**). The fold lines represent the change of the age-standardized rates (per 100,000 population) in different age groups. The shaded areas represent 95% uncertainty interval (UI) of the fold lines

For FBTs, the majority of cases occurred in the age groups between 20 and 74 years. The ASPRs for males in the age groups between 30 and 79 years were significantly higher than those for females. Although the trend in ASPR for each gender was similar, the peak occurred in the 54–59 age group for males and delayed to the 65–69 age group for females (Fig. 4a). The age-standardized DALYs rate curve showed similar trends for both genders without significant difference (Fig. 4b).

### The association between burden of zIDPs and SDI

In 2021, the ASPR of schistosomiasis decreased as the SDI increased across 204 countries and territories globally. The ASPR of cystic echinococcosis began to decline at an SDI of 0.7, while the ASPR for cysticercosis initially showed an upward trend until the SDI reached 0.6, and then started to decrease. The ASPR of FBTs exhibited a slight upward trend (Fig S4). In terms of mortality, the ASMR of schistosomiasis, cystic echinococcosis, and cysticercosis all decreased with the increase of SDI across these countries and territories (Fig. S5). In addition, the age-standardized DALYs rate of schistosomiasis, cystic echinococcosis, and cysticercosis decreased with the rise of SDI at the country scale, while no significant change observed in FBTs (Fig. S6).

**Fig. 5:**
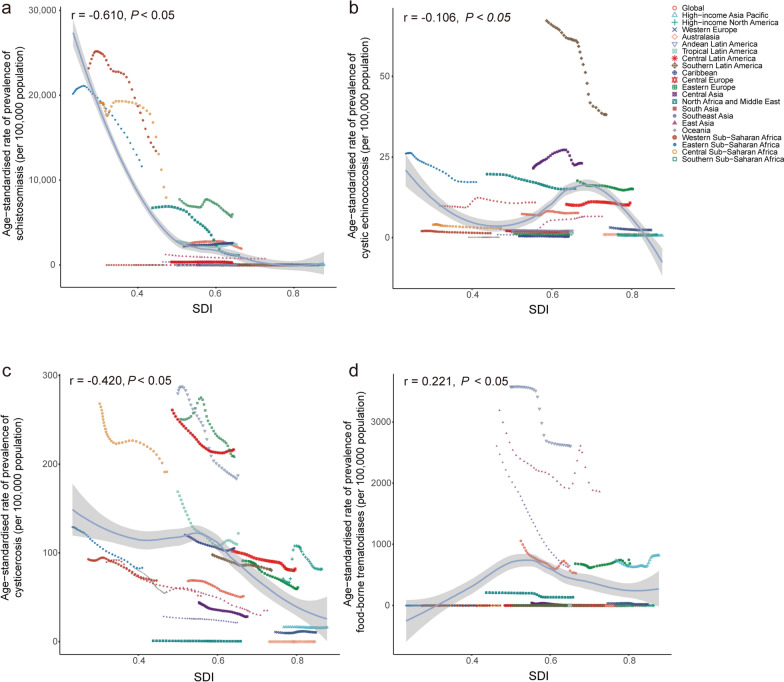
The change of age-standardized prevalence rates of schistosomiasis (**a**), cystic echinococcosis (**b**), cysticercosis (**c**), and foodborne trematodiases (**d**) with the raising SDI across all GBD regions from 1990 to 2021. SDI: Socio-demographic Index. Expected values, based on SDI and disease rates in all locations, are shown as a solid line; expected values based on a calculation accounting for the SDI and disease rates across all locations. The points are plotted for each region and show the observed age-standardized prevalence rates for each year from 1990 to 2021. The curve is derived from Spearman correlation analysis, with the shaded area representing the 95% confidence interval (*CI*). The correlation coefficient and *P* values were displayed in the top left corner of the image

From 1990 to 2021, the ASPR of schistosomiasis showed a downward trend with increasing SDI (r = − 0.610, *P* < 0.05), and the sharpest decline was observed when SDI ranged from 0.00 to 0.50. Western, Central, and Southern sub-Saharan Africa exhibited higher-than-expected ASPRs of schistosomiasis (Fig. 5a). There was no obvious association between the ASPR and SDI in cystic echinococcosis (r = − 0.106, *P* < 0.05) (Fig. 5b). For cysticercosis, ASPR decreased as SDI increased (r = − 0.4, *P* < 0.05), with a more pronounced decline after the SDI exceeded 0.50. Central and Southern sub-Saharan Africa, Andean and Central Latin America, and High-income North America exhibited higher-than-expected rates (Fig. 5c). The ASPRs of FBTs showed a slightly upward trend with rising SDI (r = 0.221, *P* < 0.05), while the higher-than-expected rates were observed in Latin America, East Asia, Southeast Asia, Eastern Europe, and high-income Asia Pacific (Fig. 5d).

**Fig. 6:**
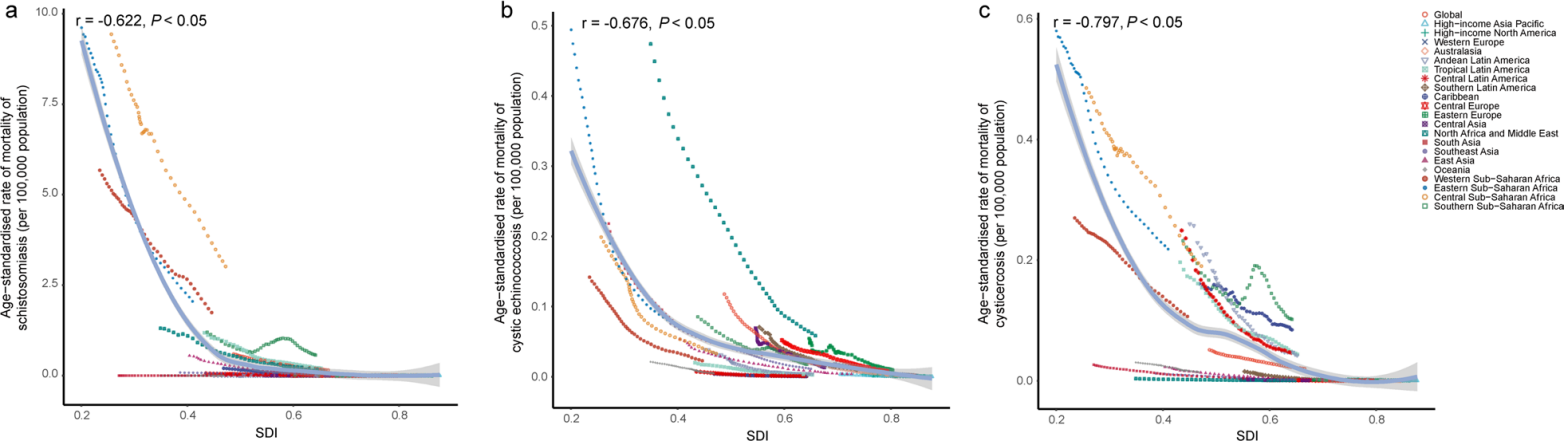
The change of age-standardized mortality rates of schistosomiasis (**a**), cystic echinococcosis (**b**), cysticercosis (**c**) with the raising SDI across all GBD regions from 1990 to 2021. SDI: Socio-demographic Index. Expected values, based on SDI and disease rates in all locations, are shown as a solid line; expected values based on a calculation accounting for the SDI and disease rates across all locations. The points are plotted for each region and show the observed age-standardized mortality rates for each year from 1990 to 2021.The curve is derived from Spearman correlation analysis, with the shaded area representing the 95% confidence interval (*CI*). The correlation coefficient and *P* values were displayed in the top left corner of the image

Regarding ASMR, schistosomiasis, cystic echinococcosis and cysticercosis all demonstrated a decrease with rising SDI (r = − 0.622, − 0.676, − 0.797, *P* < 0.05) during the period of 1990–2021, with the most pronounced decline observed in the SDI range of 0.00 to 0.50. Moreover, certain regions showed higher-than-expected ASMR, such as sub-Saharan Africa (Central and Southern) for schistosomiasis, North Africa and the Middle East for cystic echinococcosis, sub-Saharan Africa (Eastern, Central, Southern), Latin America (Andean, Tropical, Central), and the Caribbean for cysticercosis, respectively (Fig. 6).

**Fig. 7:**
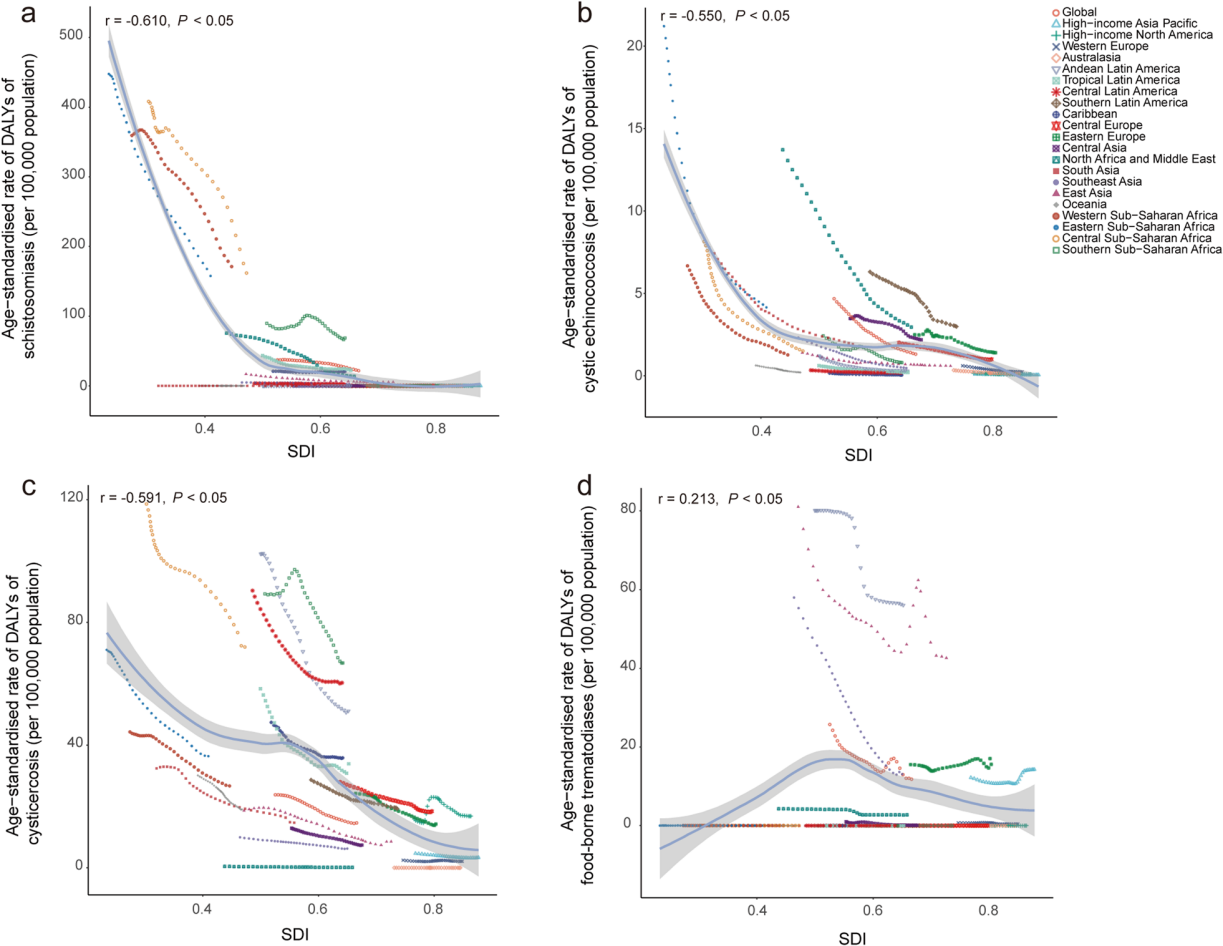
The change of age-standardized DALYs rates of schistosomiasis (**a**), cystic echinococcosis (**b**), cysticercosis (**c**), and foodborne trematodiases (**d**) with the raising SDI across all GBD regions from 1990 to 2021. SDI: Socio-demographic Index. DALYs: disability-adjusted life years. Expected values, based on SDI and disease rates in all locations, are shown as a solid line; expected values based on a calculation accounting for the SDI and disease rates across all locations. The points are plotted for each region and show the observed age-standardized DALYs rates for each year from 1990 to 2021. The curve is derived from Spearman correlation analysis, with the shaded area representing the 95% confidence interval (*CI*). The correlation coefficient and *P* values were displayed in the top left corner of the image

From 1990 to 2021, negative relationship of the age-standardized DALYs rate of schistosomiasis (r = − 0.610, *P* < 0.05), CE (r = − 0.550, *P* < 0.05) and cysticercosis (r = − 0.591, *P* < 0.05) with SDI were detected (Fig. 7a–c). However, the trends varied across the diseases and regions. The sub-Saharan Africa, North Africa and Middle East and Tropical Latin America exhibited higher-than-expected age-standardized DALY rates of schistosomiasis. South Asia, Central Asia, North Africa and Middle East, Southern Latin America and Eastern Europe had higher-than-expected age-standardized DALY rates for cystic echinococcosis (Fig. 7b). The overall age-standardized DALYs rate of cysticercosis with Central and Southern sub-Saharan Africa, Andean and Central Latin America and high-income North America had higher-than-expected age-standardized DALYs rates (Fig. 7c). In contrast, FBTs exhibited a slightly upward trend in age-standardized DALY rate with the increase of SDI (r = 0.213, *P* < 0.05). For FBTs, Latin America, East Asia, Southeast Asia, Eastern Europe, High-income Asia Pacific had higher-than-expected age-standardized DALY rates (Fig. 7d).

## Discussion

GBD 2021 is a comprehensive and updated work that assesses global health losses. It provides estimates for 371 diseases and injuries by utilizing indicators such as prevalence, incidence, years lived with disability (YLDs), years of life lost (YLLs), DALYs, and healthy life expectancy (HALE) across various age groups, genders, and locations [17]. In comparison to other diseases such as emerging infectious diseases, and cardiovascular and cerebrovascular diseases, the burden of disease caused by zIDPs might be significantly neglected, particularly during the COVID-19 pandemic [20–22]. The COVID-19 pandemic has likely diverted funding and policy attention away from zIDPs. This study presented the burden estimates of the zIDPs using GBD 2021 data, emphasizing the importance of analyzing their global burden to enhance understanding of their impacts on public health and to assess progress towards national and global control or elimination goals. Such analysis can inform policy making and effective allocation of resources, including funds, personnel, and medications.

In 2021, schistosomiasis accounted for the highest disease burden among zIDPs globally, necessitating the allocation of additional resources towards its control or elimination. According to the WHO’s 2030 roadmap for NTDs, schistosomiasis is targeted for elimination as a public health problem (currently defined as prevalence of schistosomiasis with heavy infection intensity < 1% in humans), while cystic echinococcosis, cysticercosis and FBTs are aimed at under control [20]. Although the elimination goal for schistosomiasis is ambitious, it is achievable, as demonstrated by countries like China, which have succeeded through integrated strategy focusing on management of reservoir hosts [23, 24]. Globally, the burden of these zIDPs has declined, particularly in low SDI regions, providing evidence-based support for achieving the goals set forth in the WHO roadmap of NTDs.

Based on our findings, sub-Saharan Africa loading the highest burden of schistosomiasis remains the priority region for schistosomiasis control, presenting the highest number of cases and death, the highest prevalence, mortality, and DALYs rate. East and Southeast Asia may be considered as the leading regions in achieving the WHO roadmap goals for schistosomiasis as the DALYs due to schistosomiasis significantly decreased in comparison to those in 1990s and other regions. Notably, major endemic countries like China, the Lao People’s Democratic Republic, and Cambodia had issued national elimination plans for schistosomiasis [23, 25, 26]. Furthermore, the prevalence and DALYs of schistosomiasis primarily affect individuals aged between 15 and 59, making them a critical population for intervention. As the WHO new guideline for control and elimination of human schistosomiasis issued in 2022 recommended to extend the population targeted for preventive chemotherapy to all individuals at risk of infection, more praziquantel tablets and other interventions are required to accelerate the process of schistosomiasis elimination as a public health problem by 2030 globally. In addition, the increasing of ASPR of schistosomiasis in high SDI region indicated that the disease spread from rural areas to urban due to climate change or social-economic activities. Monitoring and intervention activities also should be strengthened in these regions.

The burden of cystic echinococcosis was mainly distributed in the regions of Eastern sub-Saharan Africa, North Africa and Middle East, Southern Latin America, Central Asia and South Asia. This might be explained by the traditions of thriving livestock breeding, slaughtering, and trade activities in these regions. Notably, the burden of cystic echinococcosis (prevalence, incidence, DALYs) does not exhibit a strong correlation with SDI. Moreover, the high age-standardized DALYs and mortality rates among children aged less than 5 years enlightened a critical need to pay much attention and strengthen intervention. Furthermore, public health professionals, in collaboration with veterinarians, should prioritize promoting health of guardians, administering deworming to dogs, facilitating early diagnosis, and enhancing nutritional status [27, 28].

Unlike schistosomiasis and cystic echinococcosis, certain regions with high SDI, such as High-income North America and Eastern Europe, exhibited a notable prevalence rate of cysticercosis and associated DALYs, in addition to sub-Saharan Africa (central, southern, eastern, and western) and Latin America (central, Andean, and tropical), which remain primary contributors to the disease burden. Among the 21 GBD regions, Australasia stands out as the only region without any report on prevalence and DALYs related to cysticercosis. The absence of cysticercosis burden can likely be attributed to a series of regulations within the framework of One Health [13, 29–31], which include stringent animal quarantine and management measures for pig farming and canine animals, sophisticated healthcare infrastructure such as water supply, sanitation infrastructure, and food hygiene systems, a robust medical and veterinary service system, waste management, and strict legal oversight. In contrast, Asia especially East Asia bears the most burden of FBTs, primarily due to the regional dietary patterns, including the consumption of raw fish or other foods as a traditional or signature dish in certain areas [32, 33]. Our analysis also uncovered that the correlation between the burden of FBTs and SDI was less pronounced compared to other zIDPs, suggesting that preventing and managing these diseases could be more intricate and challenging. Therefore, in high SDI countries where raw fish and crustaceans are frequently consumed, surveillance on community members and intermediate hosts should be strengthened [34–36]. Additionally, a prolonged campaign against FBTs also should be conducted with integration of deworming, health education, behavior changes, and improving sanitation.

Despite the varying modes of infection and transmission, and differing disease burdens, all diseases pertaining to zIDPs invariably engage multiple interfaces between humans, animals, and the environment. To effectively control or eliminate zIDPs, it is crucial to underscore the One Health framework [32, 37], which highlights the importance of effective communication, collaboration, and coordination at the management level, as well as the integrated involvement of various departments and disciplines in practice. This analysis of the burden of the zIDPs offers evidence-based priorities for the future control of the zIDPs and contributes to the WHO's roadmap to end NTDs and the global One Health joint action plan.

However, this study has several limitations. First, the accuracy and completeness of the Global Burden of Disease (GBD) model estimates could be compromised by missing data from certain countries and regions [17, 20]. Second, all results in this study are derived from the GBD model rather than real-world data, which may lead to potential overestimation or underestimation. Third, the study does not account for the broader economic, familial, and social impacts on the zIDPs, highlighting the need for a more comprehensive assessment.

## Conclusions

The study retrieved and analyzed the data from GBD 2021 on the prevalence, mortality, and DALYs of the zIDPs from 1990 to 2021. This is a comprehensive characterization of the burden associated with the zIDPs to date. Meanwhile, the EAPCs of the ASPRs, ASMRs, and age-standardized DALY rates of zIDPs were calculated by age groups, sex, and SDI to illustrate the temporal changes. The results showed that the ASPR, ASMR, and age-standardized DALY rates of zIDPs declined generally from 1990 to 2021. Furthermore, these metrics exhibited distinct characteristics in terms of age, gender, GBD region and SDI. Among the zIDPs, schistosomiasis was the top disease with the highest burden of prevalence, morality, and DALYs. Regions with low and low-middle SDI should be the focus targeting for reducing the burden of zIDPs. In addition, high or high-middle regions should strengthen the surveillance to mitigate or eliminate the health risks posed by zIDPs.

## Supplementary Information

Supplementary Material 1. **Fig. S1.** The trend of global and SDI regions of age-standardized prevalence rates from 1990 to 2021. SDI: Socio-demographic Index.

Supplementary material 2. **Fig. S2.** The trend of global and SDI regions of age-standardized mortality rates from 1990 to 2021. SDI: Socio-demographic Index.

Supplementary Material 3. **Fig. S3.** The trend of global and SDI regions of age-standardized DALYs rates from 1990 to 2021. DALYs: disability-adjusted life years. SDI: Socio-demographic Index.

Supplementary Material 4. **Fig. S4.** The trend of age-standardized prevalence rates of schistosomiasis (a), cystic echinococcosis (b), cysticercosis (c), and foodborne trematodiases (d) with raising SDI across 204 countries and territories. SDI: Socio-demographic Index.

Supplementary Material 5. **Fig. S5.** The trend of age-standardized mortality rates of schistosomiasis (a), cystic echinococcosis (b), and cysticercosis (c) with raising SDI across 204 countries and territories. SDI: Socio-demographic Index.

Supplementary Material 6. **Fig S6.** The trend of age-standardized DALYs rates of schistosomiasis (a), cystic echinococcosis (b), cysticercosis (c), and foodborne trematodiases (d) with raising SDI across 204 countries and territories. SDI: Socio-demographic Index.

Supplementary Material 7. **Table S1.** The estimated number cases of zoonotic infectious diseases of poverty in 1990 and 2021,and estimated annual changes from 1990 to 2021. *EAPCs* estimated annual percentage changes, *SDI* Socio-demographic Index, *UI* uncertainty intervals; *CI* confidence interval.

Supplementary Material 8. **Table S2.** The estimated number of deaths of zoonotic infectious diseases of poverty in 2021, and estimated annual changes from 1990 to 2021. *EAPCs* estimated annual percentage changes, *SDI* Socio-demographic Index, *UI* uncertainty intervals; *CI* confidence interval*.*

Supplementary Material 9. **Table S3.** The estimated number of DALYs of zoonotic infectious diseases of poverty in 2021, and estimated annual changes from 1990 to 2021. *EAPCs* estimated annual percentage changes, *SDI* Socio-demographic Index, *UI* uncertainty intervals; *CI* confidence interval.

## Data Availability

Not applicable.

## Abbreviations

ASMR: Age-standardized mortality rate
ASPR: Age-standardized prevalence rate
ASRs: Age-standardized rates
*CI*: Confidence interval
COVID-19: Coronavirus disease 2019
DALY: Disability-adjusted life year
DisMoD-MR: Disease-model-Bayesian meta-regression
EAPC: Estimated annual percentage change
FBTs: Food-borne trematodiases
GATHER: Guidelines for Accurate and Transparent Health Estimates Reporting
GBD: Global Burden of Diseases, Injuries, and Risk Factors Study
GHDx: Global Health Data Exchange
IHME: Institute for Health Metrics and Evaluation
SDI: Sociodemographic Index
UI: Uncertainty interval
WHO: World Health Organization.
zIDPs: Zoonotic infectious diseases of poverty
