# Supplementary figures and images for: Global burden of zoonotic infectious diseases of poverty, 1990–2021
Source: Infect Dis Poverty. 2024 Nov 6;13:82. doi: 10.1186/s40249-024-01252-x (PMC11539463; doi:10.1186/s40249-024-01252-x)

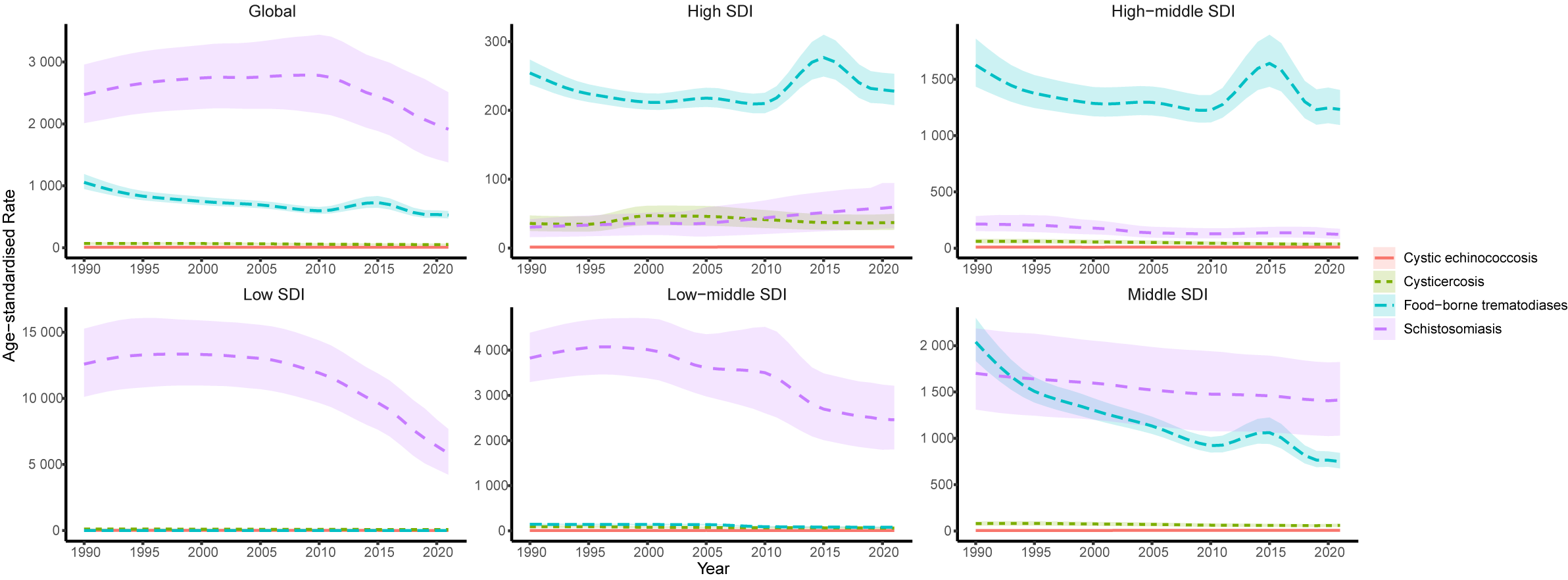

Supplement: Supplementary file 1 — Supplementary Material 1. Fig. S1. The trend of global and SDI regions of age-standardized prevalence rates from 1990 to 2021. SDI: Socio-demographic Index. [file 40249_2024_1252_MOESM1_ESM.png]

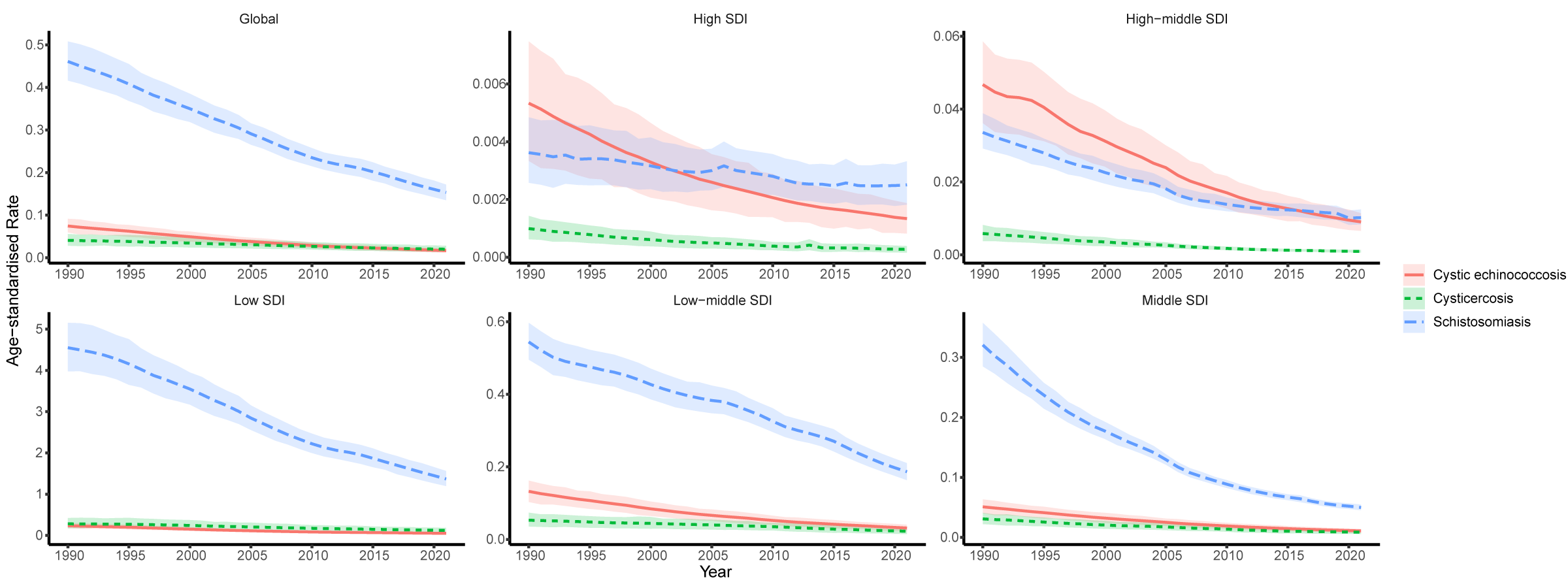

Supplement: Supplementary file 2 — Supplementary material 2. Fig. S2. The trend of global and SDI regions of age-standardized mortality rates from 1990 to 2021. SDI: Socio-demographic Index. [file 40249_2024_1252_MOESM2_ESM.png]

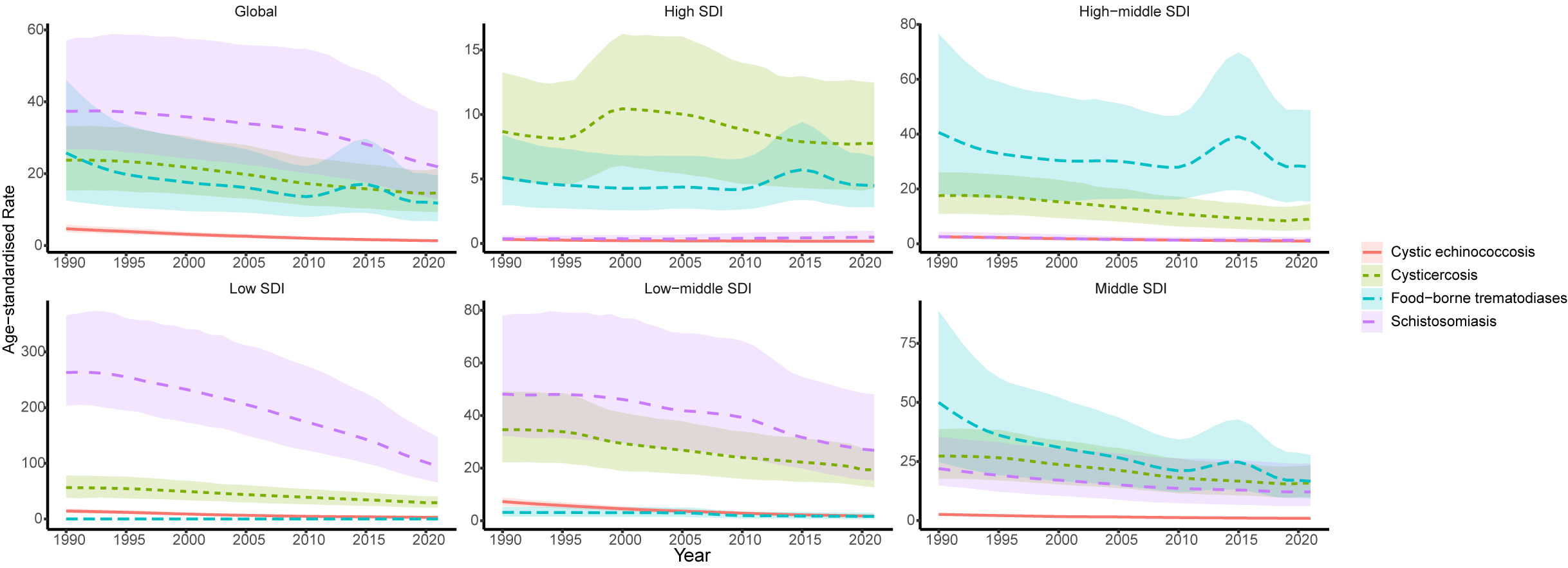

Supplement: Supplementary file 3 — Supplementary Material 3. Fig. S3. The trend of global and SDI regions of age-standardized DALYs rates from 1990 to 2021. DALYs: disability-adjusted life years. SDI: Socio-demographic Index. [file 40249_2024_1252_MOESM3_ESM.png]

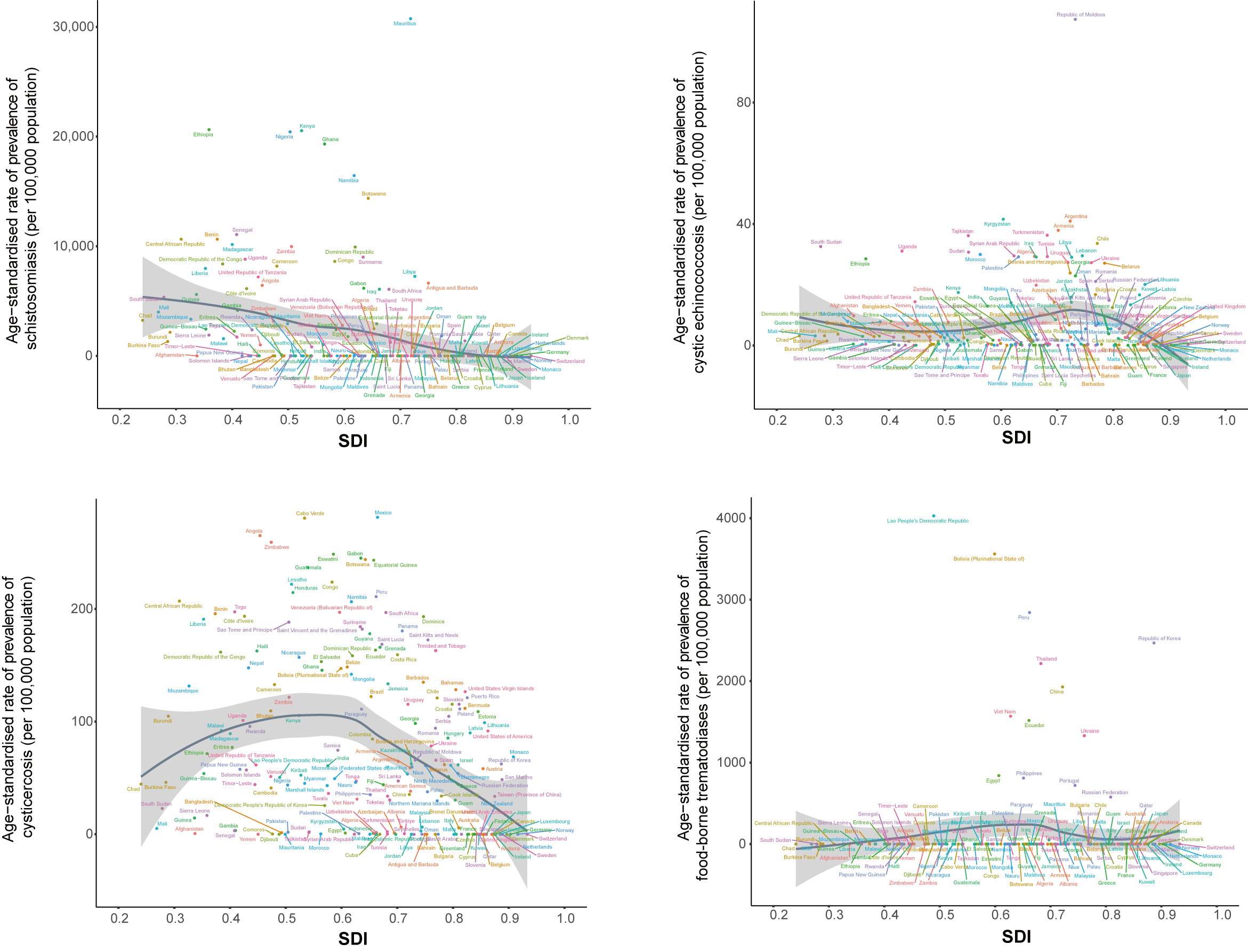

Supplement: Supplementary file 4 — Supplementary Material 4. Fig. S4. The trend of age-standardized prevalence rates of schistosomiasis (a), cystic echinococcosis (b), cysticercosis (c), and foodborne trematodiases (d) with raising SDI across 204 countries and territories. SDI: Socio-demographic Index. [file 40249_2024_1252_MOESM4_ESM.png]

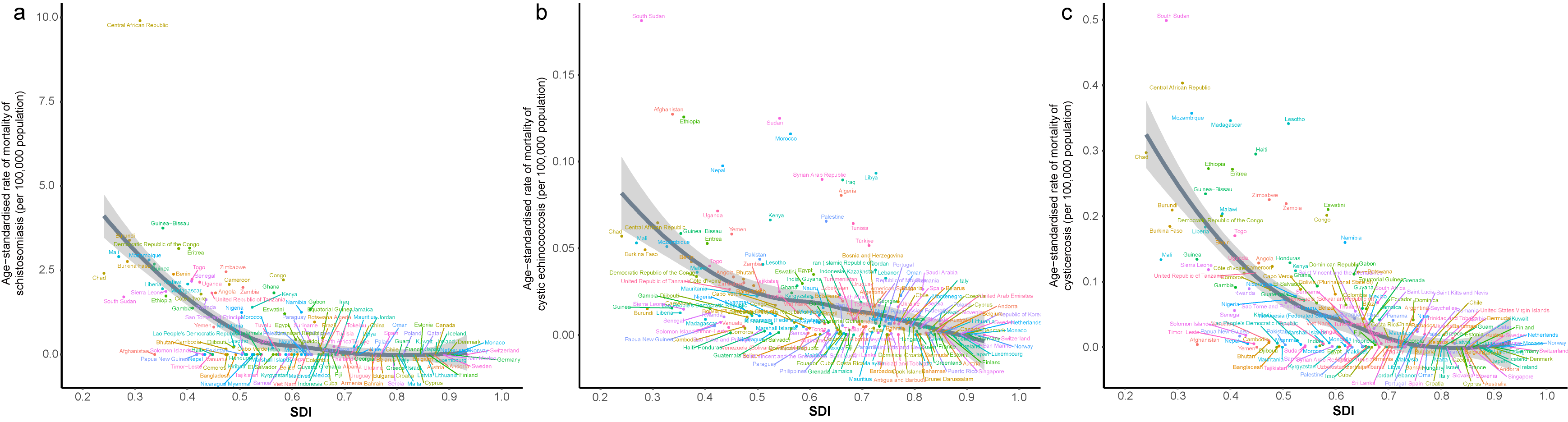

Supplement: Supplementary file 5 — Supplementary Material 5. Fig. S5. The trend of age-standardized mortality rates of schistosomiasis (a), cystic echinococcosis (b), and cysticercosis (c) with raising SDI across 204 countries and territories. SDI: Socio-demographic Index. [file 40249_2024_1252_MOESM5_ESM.png]

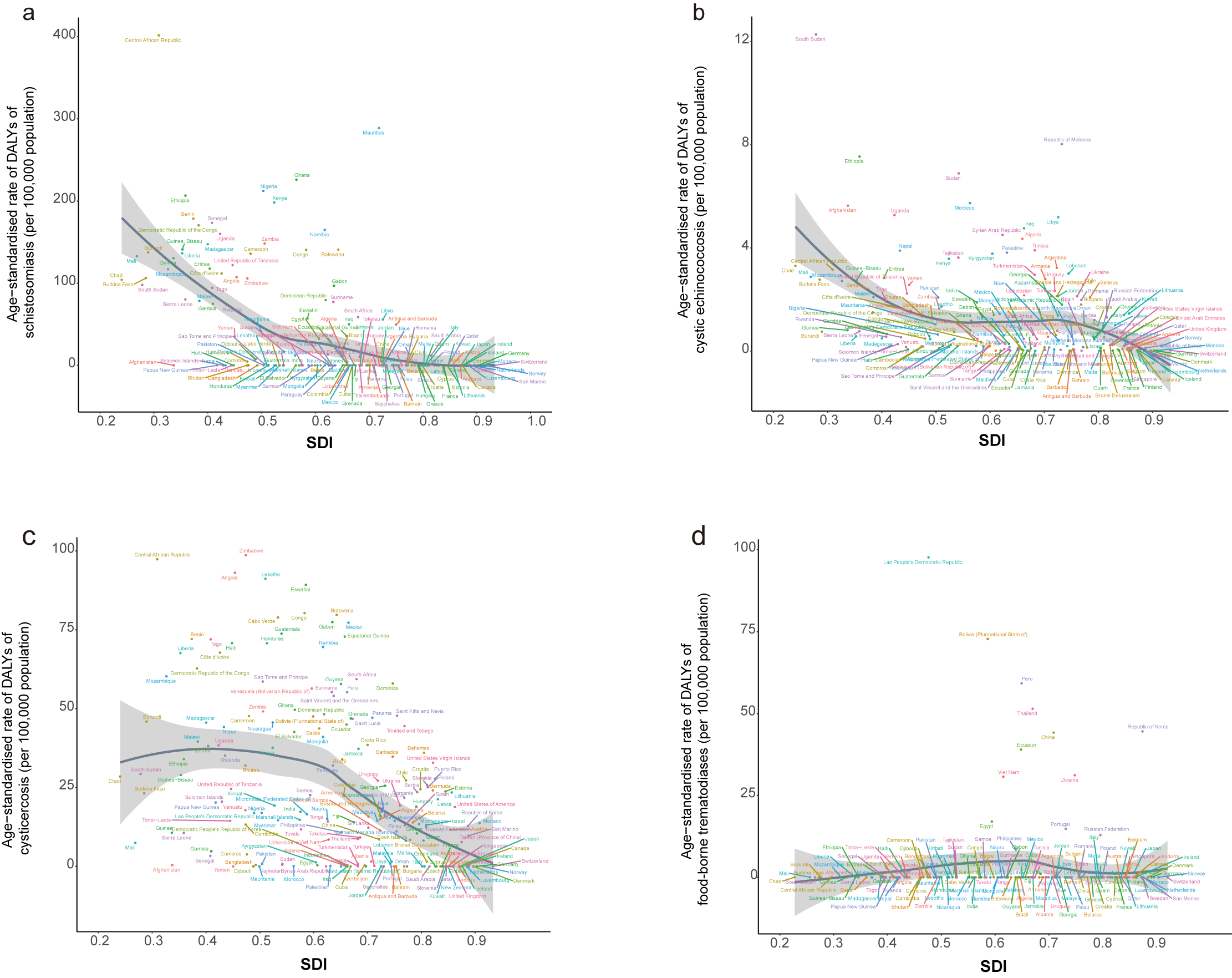

Supplement: Supplementary file 6 — Supplementary Material 6. Fig S6. The trend of age-standardized DALYs rates of schistosomiasis (a), cystic echinococcosis (b), cysticercosis (c), and foodborne trematodiases (d) with raising SDI across 204 countries and territories. SDI: Socio-demographic Index. [file 40249_2024_1252_MOESM6_ESM.png]
